# Effects of two gait retraining programs on pain, function, and lower limb kinematics in runners with patellofemoral pain: A randomized controlled trial

**DOI:** 10.1371/journal.pone.0295645

**Published:** 2024-01-10

**Authors:** José Roberto de Souza Júnior, Pedro Henrique Reis Rabelo, Thiago Vilela Lemos, Jean-Francois Esculier, Glauber Marques Paraizo Barbosa, João Paulo Chieregato Matheus

**Affiliations:** 1 Graduate Program of Sciences and Technologies in Health, University of Brasília, Brasília, Federal District, Brazil; 2 Moving Physical Therapy, Goiânia, Goiás, Brazil; 3 Department of Physical Therapy, State University of Goiás, Goiânia, Goiás, Brazil; 4 The Running Clinic, Lac Beauport, Quebec, Canada; 5 Department of Physical Therapy, University of British Columbia, Vancouver, British Columbia, Canada; 6 Graduate Program of Sciences in Health, Federal University of Goiás, Goiânia, Goiás, Brazil; The Hong Kong Polytechnic University, HONG KONG

## Abstract

**Background:**

Patellofemoral Pain (PFP) is one of the main injuries in runners. Consistent evidence support strengthening programs to modulate symptoms, however, few studies investigated the effects of gait retraining programs.

**Objective:**

To investigate the effects of two different two-week partially supervised gait retraining programs on pain, function, and lower limb kinematics of runners with PFP.

**Methods:**

Randomized controlled trial. Thirty runners were allocated to gait retraining groups focusing on impact (n = 10) or cadence (n = 10), or to a control group (n = 10). Impact group received guidance to reduce tibial acceleration by 50%, while cadence group was asked to increase cadence by 7.5–10%. The control group did not receive any intervention. Usual and running pain, knee function, and lower limb kinematics (contralateral pelvic drop, hip adduction, knee flexion, ankle dorsiflexion, tibia inclination, and foot inclination) were evaluated before (T_0_), immediately after the intervention (T_2_), and six months after the protocol (T_24_).

**Results:**

A significant group x time interaction was found for running pain (p = 0.010) and knee function (p = 0.019). Both programs had greater improvements in running pain compared to no intervention at T_24_ (Impact x Control—mean difference (MD) −3.2, 95% CI −5.1 to −1.3, p = 0.001; Cadence x Control—MD −2.9, 95% CI −4.8 to −1.0, p = 0.002). Participants of the impact group had greater improvements in knee function compared to no intervention at T_2_ (Impact x Control–MD 10.8, 95% CI 1.0 to 20.6, p = 0.027). No between-group differences in usual pain and lower limb kinematics were found (p>0.05).

**Conclusion:**

Compared to no intervention, both programs were more effective in improving running pain six months after the protocol. The program focused on impact was more effective in improving knee function immediately after the intervention.

**Clinical trial registry number:** RBR-8yb47v

## Introduction

Patellofemoral pain (PFP) can be defined as pain around or behind the patella during functional activities that load the patellofemoral joint (e.g., jogging, running, squatting, hopping/jumping) [1]. It has a prevalence of 29% in adolescents [2], 23% in the general population [2], and 5.5% in runners [3], being one of the main overuse injuries in this sport. In addition, the poor long-term prognosis must be highlighted, 55% of the patients experience unfavorable recovery over 3 months [4], 40% over 12 months [4], and 57% over 5 to 8 years [5].

Interventions that focus on hip and knee-targeted exercises and running biomechanics have been used to manage patients with PFP [6, 7]. Consistent evidence supports that exercise therapy has positive effects on usual pain, pain during activity, and functional ability in the short and long term compared to a control strategy (e.g. no intervention, placebo) [6]. However, only one randomized controlled trial (RCT) provided evidence about the effects of changes in running technique (gait retraining) on clinical outcomes compared with no intervention in runners with PFP [8]. A two-week supervised program to change the foot strike pattern from rearfoot strike to forefoot strike was applied and runners with PFP presented a reduction in usual pain immediately and one month after the protocol [8].

Unfortunately, the strategy used may not be suitable for all participants. Transitioning to a non-rearfoot strike was associated with reduced peak knee flexion, knee flexion excursion, peak knee extensor moment, and patellofemoral joint (PFJ) stress [9]; at the same time, increases in ankle excursion, plantar flexor moment, eccentric power, negative work, and axial contact force were also reported [9, 10]. These findings suggest that a rapid increase in absorbed ankle energy could represent a potential risk for ankle injuries, especially at the Achilles tendon [10]. In fact, two runners changing to a forefoot strike (25%) in a previous RCT reported ankle soreness at the one-month follow-up [8].

The use of feedback to decrease tibial acceleration [11, 12] and to increase cadence [13–17] may be safer alternatives. A two-week supervised program with feedback on tibial acceleration reduced loading rates in healthy subjects immediately after the protocol [11, 12] and results were maintained through one year [12]. Also, a 62% lower occurrence of running injury was found after a two-week supervised gait retraining program with visual feedback on average vertical loading rate [18]. While PFP was one of the main injuries in the control group (29%), participants that performed the gait retraining program presented mainly Achilles tendinitis (18%) and calf strain (18%) [18].

About cadence, an increase of 5–10% promoted a reduction in peak knee flexion, knee work, peak knee extensor moment, and peak PFJ stress [19]. Additionally, a reduction in variables of the hip (i.e., peak hip adduction and hip work) and ankle (i.e., foot strike angle, gastrocnemius peak force, and ankle work) indicate that the demand was not shifted to these joints [19, 20]. Gait retraining programs that increased cadence by 7.5–10% reduced pain levels post-training [13–17], and results were maintained six-months [17] after the protocol. These findings show the potential use of these strategies that need to be tested against a control intervention in runners with PFP.

Despite the gait retraining modality chosen, the format must be feasible for clinicians; the classic protocols use a faded feedback design with eight supervised sessions over two weeks with a duration of 15 to 30 minutes [8, 11, 12]. This protocol prevents dependency on external feedback and generates long-term retention [21], however, it is challenging to replicate in clinical practice given the time and financial constraints for patients [22].

In order to address the lack of RCTs that tests the efficacy of gait retraining programs with better external validity, our goal was to investigate the effects of two different two-week partially supervised gait retraining programs, one focusing on impact and the other focusing on cadence, with a control intervention; on pain, function and lower limb kinematics of runners with PFP. Our hypothesis was that both partially supervised gait retraining programs would be more effective in reducing pain, improving symptoms, and modifying lower limb kinematics during running compared with the control group, and that the positive effects from these programs would persist for six months. Also, we believed that one gait retraining group would not be superior to the other.

## Materials and methods

### Trial design

**Fig 1:**
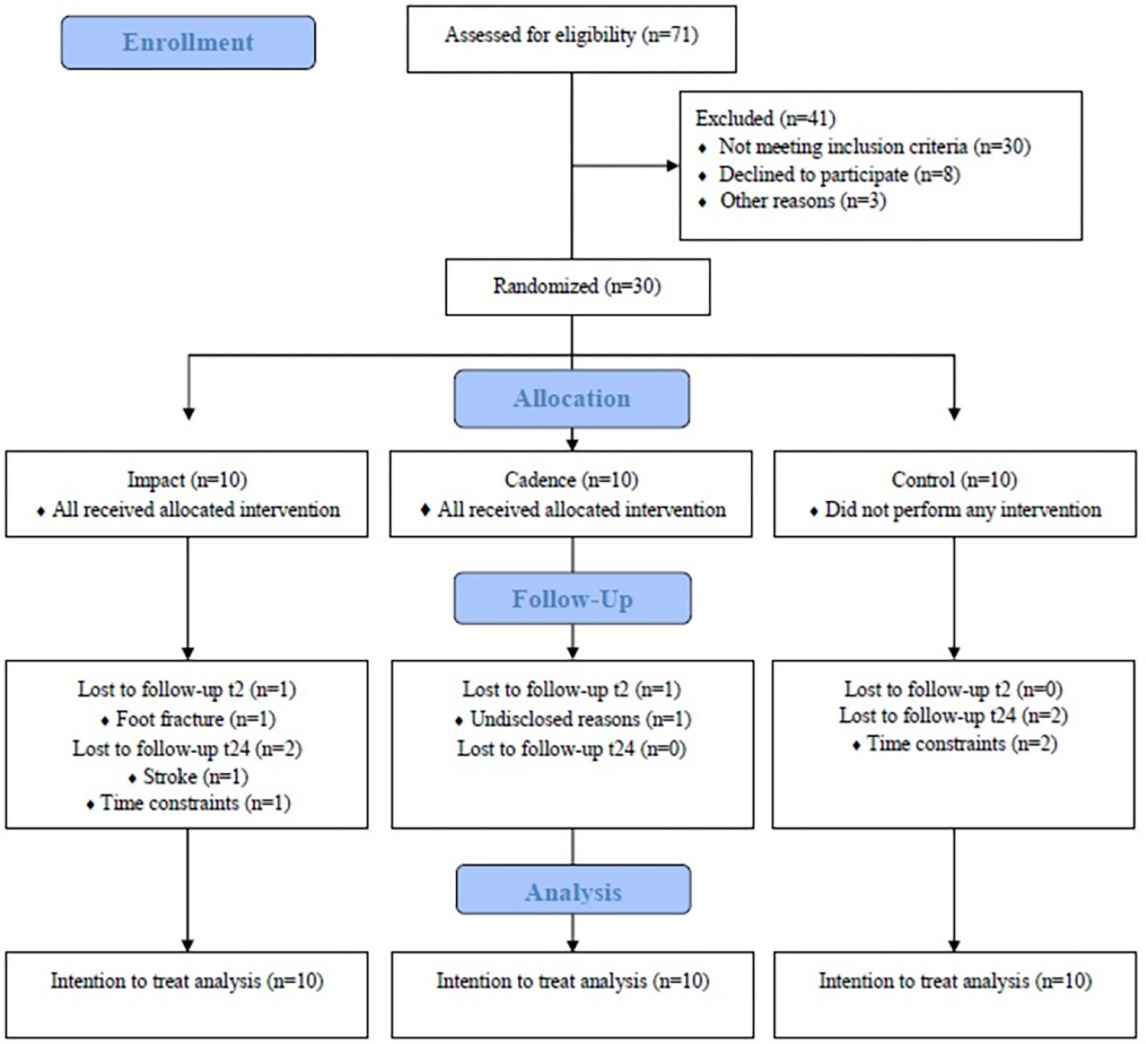
Flow diagram of the study.

This is a randomized, single-blind, parallel group, three-arm superiority clinical trial, using a 1:1:1 allocation rate and six-month follow-up. The study was composed of three groups: a group that performed partially supervised gait retraining with a focus on impact, a group that performed partially supervised gait retraining with a focus on cadence, and a control group that did not perform any intervention. The outcomes were evaluated before (T_0_), immediately after (T_2_), and six months after the protocol (T_24_). Ethics approval was obtained from the Institutional Review Board of the Ceilândia Faculty, University of Brasília (Approval number: 22631019.7.0000.8093, 07/03/2020), and all subjects signed a detailed consent form before entering the study. This research was prospectively registered at the Brazilian Registry of Clinical Trials (RBR-8yb47v) and was reported in accordance with the CONSORT (Consolidated Standards Of Reporting Trials) (Fig 1). The detailed protocol of this trial was published elsewhere [23].

### Participants

Participants were recruited using advertisements within the running community of Goiânia, Brazil. The trial was conducted at the Instituto Trata, Goiânia, Brazil between August 2020 and July 2022. We included male and female runners, aged between 18 and 45 years, that presented pain around or behind the patella, with intensity of at least 3/10 on a visual analogue scale (VAS), during running and one task among squatting, climbing, and descending steps, kneeling and extending the knee with resistance [1], and that were able to run on a treadmill at a speed of 10–12 km/hour for six minutes. To not include participants with Patellofemoral Osteoarthritis the limit of 45 years old was used. Participants were excluded if they presented other disorders in the lower limbs or history of surgery in the lower limbs in the last year. These aspects may affect running biomechanics. Also, participants were excluded if no interest to adhere to a strict running retraining protocol for 2 weeks was reported. It is important to outline a change in the eligibility criteria compared with the original protocol; non-rearfoot strike runners with a cadence greater than 170 steps/min were included to facilitate recruitment. No differences in spatiotemporal aspects exist between rearfoot and non-rearfoot strikers; however, differences in the kinematics of the lower limbs and loading rates were described [24]. Also, the aforementioned value was used in previous studies as cut-off points for lower cadence [25].

### Randomization and allocation concealment

Participants were randomized into experimental or control groups with an allocation ratio of 1:1:1 by means of block randomization (block size of 15) performed with the aid of a sequence of numbers generated on a computer using the website www.randomizer.org. The allocation was hidden by means of opaque envelopes, sealed, and numbered consecutively. A laboratory employee who did not participate in the evaluations and interventions generated the allocation sequence, hid the allocation, and allocated participants for interventions.

### Blinding

The researcher who collected data and performed assessments was blinded to group allocation. Due to the nature of the intervention, both the participants and the researcher responsible for the gait retraining could not be blinded; however, they were strongly warned not to reveal the allocation in subsequent evaluations and instructed to perform the retraining sessions alone. In this way, the risk of the blinded-assessor and other participants knowing what retraining is being performed was diminished.

### Interventions

Before starting the intervention, participants ran with their regular shoes [26] at a speed of 10 km/hour, in order to verify the usual values of the vertical tibial acceleration and cadence. After this assessment, it was possible to establish the threshold of the vertical tibial acceleration and cadence that were used in the programs. Both parameters were acquired with the accelerometer Tgforce (v2.0.0.10) [27] taped to the anteromedial aspect of the subject’s distal tibia [11].

In the experimental groups, the protocol was performed four times a week, with a gradual duration from 15 to 30 minutes, over two weeks. There were two supervised (first and fifth session) and six unsupervised sessions. In the final 4 training sessions the feedback was gradually removed.

**Fig 2:**
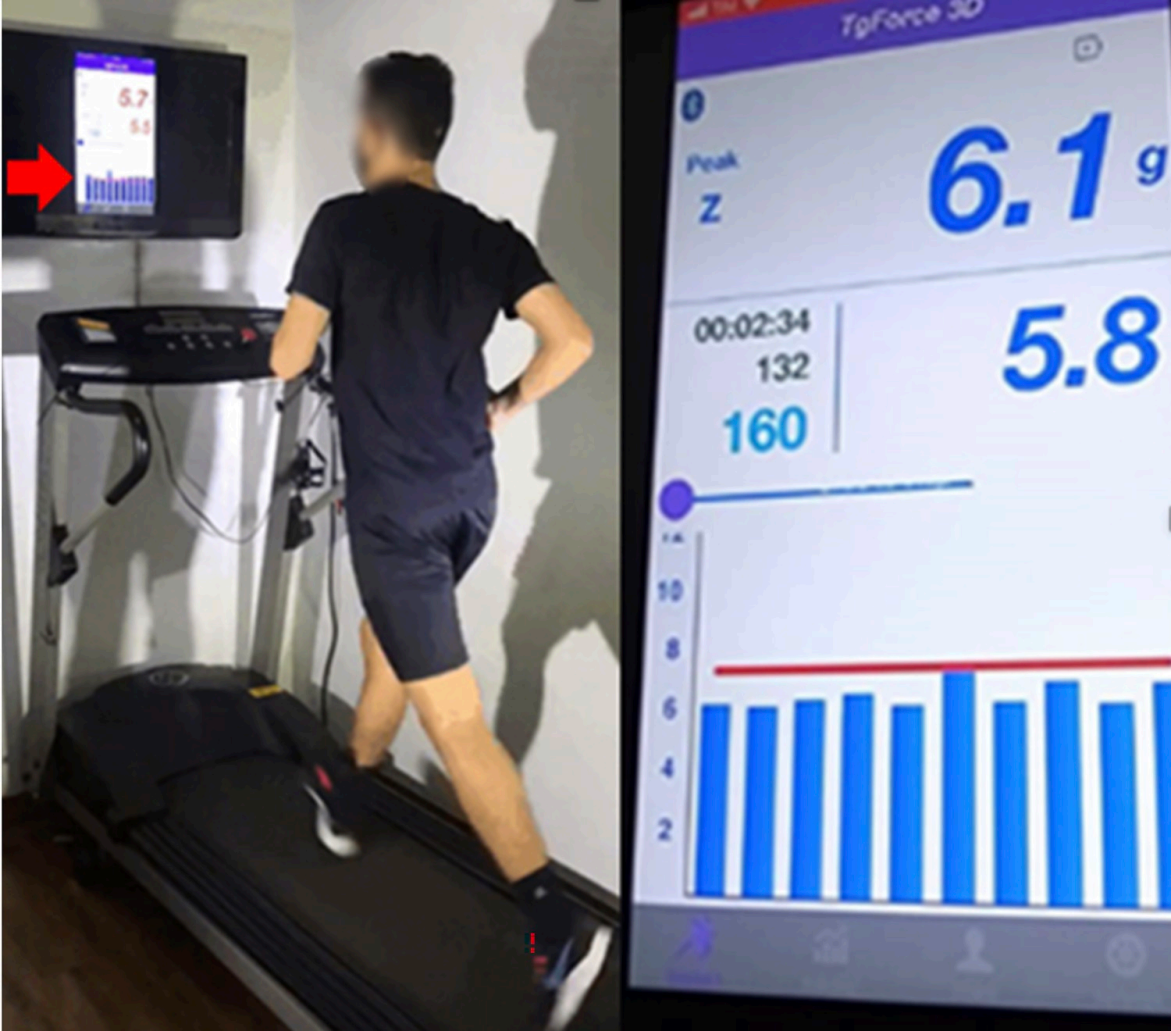
Visual feedback provided to impact group participants.

Impact group participants received visual feedback (vertical tibial acceleration captured using the accelerometer) and verbal feedback (commands given by the clinician) during the supervised sessions. A screen positioned in front of the treadmill showed a graph of real-time tibial acceleration captured by the accelerometer. On the screen, the participant saw a line representing approximately 50% of the average peak tibial acceleration obtained during the baseline assessment [11, 12]. Subjects were instructed to “run softer,” “make their footfalls quieter”, and to keep the acceleration peaks below the line [11, 12, 28] (Fig 2).

During the unsupervised sessions participants received audio feedback through the Tgforce. A beep was generated when the acceleration values exceeded 50% of the threshold value predetermined. Subjects were instructed to “run softer,” “make their footfalls quieter” and to run without any beeps. Cadence group participants received guidance regarding their cadence and ran with the help of a metronome with an adjusted cadence increased by 7.5 to 10% [20, 22, 29] during the supervised and unsupervised sessions. In both groups, the unsupervised sessions were performed at a location of their choice, as long as it was overground running. In both groups, participants performed the retraining sessions using a comfortable speed. In addition, participants were instructed to run with this new running-pattern during the following six months. The adverse effects were collected and addressed descriptively in the study results. Control group participants did not receive any retraining strategies or guidance until the end of the six-month follow-up period.

### Outcomes

Initially, participants were screened to confirm eligibility. Participants’ characteristics (gender, age, body mass, height, body mass index, experience, training volume, and training frequency) were collected at baseline assessment. Primary and secondary outcomes were measured at the baseline assessment, immediately after the two-week intervention period, and six months after the protocol. The primary outcomes were usual pain and pain during running [30]. The secondary outcomes were knee function [31], and the kinematics of the lower limbs in the frontal (contralateral pelvic drop; hip adduction) [32–34] and sagittal planes (knee flexion; ankle dorsiflexion; tibia inclination; foot inclination) [32, 35]. Vertical tibial acceleration and cadence were collected as described previously at the same follow-up time points.

Usual pain and running pain were assessed using the VAS [30], which consists of a numerical scale from 0 to 10 points, where 0 means no pain and 10 means the maximum pain ever experienced. Knee function was assessed using the Patellofemoral Disorders Scale (Kujala Scale) [31], which contains 13 questions that assess the severity of symptoms and limitations in different activities related to PFP. It presents a score between 0 to 100 points where the lower the score the worse the function. Usual pain assessed using the VAS and knee function assessed using the Kujala scale are reliable, valid, and responsive measures recommended for clinical trials performed with patients with PFP [36].

**Fig 3:**
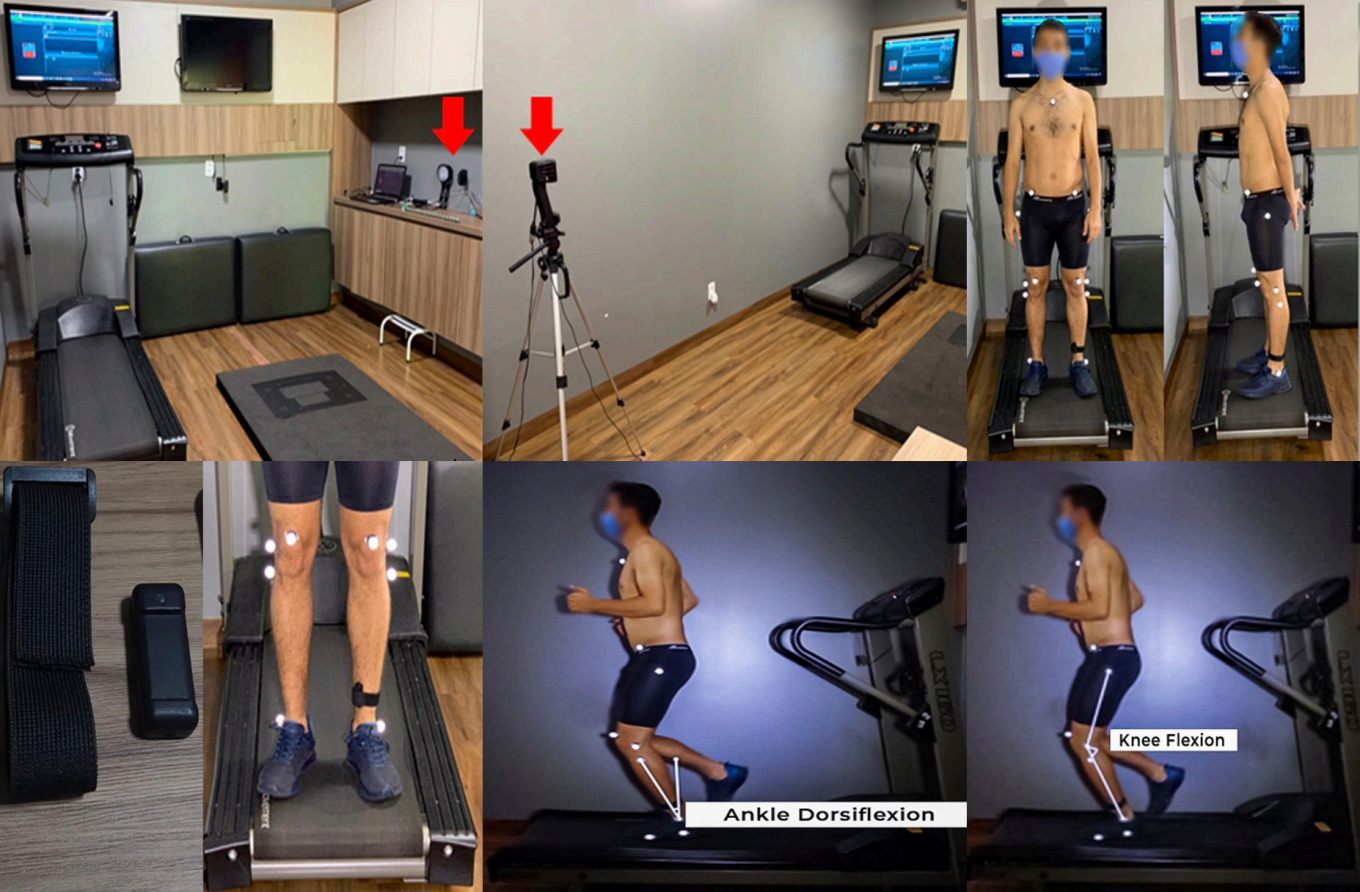
Experimental setup and an example of the two-dimensional measurements.

Lower limb kinematics were assessed using two webcams (MyoVideo 139 HD Color Webcam) sampling at 30 frames per second and two leds (LED Floodlight) [37]. Reflective markers were placed on the manubrium sterni and bilaterally on the anterior superior iliac spine (ASIS), greater trochanter, lateral femoral epicondyle, fibular head, and lateral malleolus [32]. An additional marker was placed at the fifth metatarsal. All participants were instructed to run at 10 km/hour on a motorized treadmill (Movement XL 1600). The peaks in degrees of the contralateral pelvic drop and hip adduction were evaluated at midstance [32–34]. The peaks in degrees of knee flexion and ankle dorsiflexion were evaluated at midstance, while tibial and foot inclination were evaluated at initial contact [32, 35]. The video recordings were analyzed using the software MyoResearch 3.14—MyoVideo (Noraxon U.S. A. Inc.) (Fig 3). To analyze the proposed angles, seven steps were considered. A previous study showed that this was the number of steps needed to reach and maintain a stable mean [32].

### Data analysis

A priori sample size calculation was conducted using the Eta-partial square value (η2) of the group-by-time interaction for worst knee pain (η2 = 0.19) obtained in a previous study that compared the effects of three gait retraining programs on runners with patellofemoral pain [17]. In G*Power software [38] we used repeated-measures ANOVA, within-between interaction, with the following parameters: effect size f = 0.48 (obtained using η2 = 0.19); level of significance = 0.05; power = 99%; number of groups = 3; number of measurements = 3. The G*Power software uses the effect size index (f) for this analysis. The effect size f was calculated directly using the η2 through the following formula: f = √η2/(1−η2). The total sample size obtained was 21 participants; however, to account for losses during the follow-up period, the final sample size was 30 participants.

Data were analyzed using SPSS (Statistical Package for Social Sciences) version 26.0. Descriptive statistics consisted in means and standard deviation for continuous variables and frequencies and percentages for categorical variables. Data normality was tested using the Shapiro-Wilk test, while sphericity was tested using Mauchly’s test. Participant’s characteristics were compared using one-way ANOVA (parametric data), Kruskal-Wallis (non-parametric data) and Fisher’s exact test (categorical variables). Between-group differences (treatment effects) and their 95% confidence intervals (CIs) were calculated using repeated-measures ANOVAs (group x time). Group (impact x cadence x control) was used as the independent factor, time (T_0_ x T_2_ x T_24_) as the repeated factor, and the primary and secondary outcomes as dependent variables. Sidak’s posthoc was used to make pairwise comparisons. A statistical significance level of p<0.05 was chosen. Effect sizes were determined using generalized eta-squared (η^2^G) for the ANOVAs. Values of η^2^G>0.01 were defined as small, η^2^G>0.06 as medium, and η^2^G>0.14 as large [39]. Hedges’s g (*g*) was used for the pairwise comparisons. Values of *g* = 0.2 were defined as small, *g* = 0.5 as medium, and *g* = 0.8 as large [39]. An intention-to-treat analysis was performed for all randomized participants. Missing data were replaced using multiple imputations. Subgroup analyses were not performed.

## Results

### Recruitment and baseline data

From a total of 71 runners assessed for eligibility, 30 were recruited and assigned to one of the three study groups between August 2020 and January 2022. At T_2_, one participant from the impact group (follow-up rate = 90%) dropped out due to a traumatic foot fracture non-related to the gait retraining program, and one participant from the cadence group (follow-up rate = 90%) dropped out due to undisclosed reasons. No biomechanical data were collected for one runner at T_2_ because of a lateral ankle sprain sustained during the week of the assessment. At T_24_, two additional participants from the impact group (follow-up rate = 70%) dropped out due to a stroke and time constraints, and two dropped out from the control group due to time constraints (follow-up rate = 80%). No biomechanical data were collected for one runner of the control group and one runner of the cadence group at T_24_ because of time constraints at the week of the assessment. As for adverse effects, two participants of the impact group reported minor problems; one reported calf soreness, while the other reported bilateral upper trapezius discomfort. These problems did not affect the retraining programs.

The groups presented similar distributions for gender, mean age of 30 years, and runners of the control group were heavier Table 1. Most participants had been running for more than one year, with a low volume, and a frequency of three times per week. Patients began the study with moderate levels of pain and disability, and presented similar kinematics and spatio-temporal data. No between-group differences were found across the groups for the general characteristics, pain, knee function, and running biomechanics (all p>0.05) Table 1.

**Table 1:** Baseline participants characteristics (n = 30).

Outcomes	Impact (n = 10)	Cadence (n = 10)	Control (n = 10)	P
**Demographics**				
**Sex**				0.873
**Male**	5 (50%)	6 (60%)	6 (60%)	
**Female**	5 (50%)	4 (40%)	4 (40%)	
**Age (years)**	32.1 (5.8)	30.2 (5.3)	29.0 (4.4)	0.426
**Weight (kg)**	72.3 (13.3)	73.7 (20.1)	81.6 (20.9)	0.487
**Height (m)**	1.70 (0.1)	1.74 (0.1)	1.72 (0.1)	0.717
**Body Mass Index (kg/m^2^)**	24.7 (2.2)	23.8 (3.7)	27.1 (4.8)	0.169
**Training**				
**Running experience**				0.321
**>1 year**	8 (80%)	10 (100%)	7 (70%)	
**<1 year**	2 (20%)	0 (0%)	3 (30%)	
**Running distance (km/week)**	20.6 (15.2)	12.1 (12.6)	15.3 (9.9)	0.170
**Running frequency (times/week)**	3.0 (0.8)	3.0 (1.6)	3.1 (1.1)	0.766
**Pain and knee function**				
**Usual pain (0–10)**	3.5 (2.2)	4.5 (2.3)	4.4 (2.2)	0.607
**Running pain (0–10)**	5.4 (1.5)	6.1 (1.1)	5.6 (2.1)	0.657
**Kujala scale (0–100)**	82.7 (6.9)	84.6 (7.8)	76.6 (12.2)	0.152
**Running Biomechanics**				
**VTA (g)**	7.1 (2.4)	6.3 (1.6)	7.0 (1.8)	0.602
**Cadence (steps/min)**	172.4 (9.9)	167.6 (6.4)	168.4 (7.1)	0.365
**CPD (°)**	4.7 (2.8)	5.2 (1.5)	6.4 (3.5)	0.181
**Hip adduction (°)**	75.3 (4.7)	78.4 (3.2)	76.1 (6.4)	0.363
**Knee flexion (°)**	43.9 (4.6)	42.3 (5.1)	42.3 (4.5)	0.612
**Ankle dorsiflexion (°)**	27.4 (4.6)	26.7 (3.5)	25 (2.8)	0.460
**Tibia inclination (°)**	9.5 (2.6)	7.2 (4.4)	7.7 (2.9)	0.393
**Foot inclination (°)**	11.4 (4.4)	8.3 (8.6)	11.5 (4.2)	0.420

Categorical variables are expressed as number (%). Continuous variables are expressed as mean (SD). VTA, Vertical Tibial Acceleration. CPD, Contralateral Pelvic Drop. The peaks in degrees of knee flexion and ankle dorsiflexion were evaluated at midstance, while tibial and foot inclination were evaluated at initial contact.

### Pain and knee function

A significant group x time interaction and a medium effect size were found for running pain (p = 0.010, η^2^G = 0.10) and knee function (p = 0.019, η^2^G = 0.09). Both intervention groups had greater improvements in running pain, with a large effect size, compared to the control group at T_24_ (Impact x Control—mean difference (MD) −3.2, 95% CI −5.1 to −1.3, p = 0.001, *g* = -2.34; Cadence x Control—MD −2.9, 95% CI −4.8 to −1.0, p = 0.002, *g* = -1.66). Patients allocated to the impact group had greater improvements in knee function, with a large effect size, compared to the control group at T_2_ (Impact x Control–MD 10.8, 95% CI 1.0 to 20.6, p = 0.027, *g* = 1.22). No significant group x time interaction and a small effect size were found for usual pain (p = 0.127, η^2^G = 0.05) Table 2.

**Table 2:** Mean (SD) and mean difference and 95% CIs for pain and function outcomes (n = 30).

	Mean (SD)					Between-group difference (95% CI)		
**Outcomes**	**Impact**	**Cadence**	**Control**	**P**	**η** ^**2**^ **G**	**Impact x**	**Cadence x**	**Impact x**
						**Control**	**Control**	**Cadence**
**Usual Pain (0–10)**								
**T**_**0**_	3.5 (2.2)	4.5 (2.3)	4.4 (2.2)			-0.9 (-3.4 to 1.7)	0.1 (-2.5 to 2.6)	-1.0 (-3.5 to 1.6)
**T**_**2**_	0.7 (0.7)	1.3 (1.2)	3.5 (2.4)	0.127	0.05	-2.8 (-4.6 to -0.9)	-2.2 (-4.1 to -0.3)	-0.6 (-2.4 to 1.2)
**T**_**24**_	0.5 (0.6)	1.0 (1.4)	2.0 (2.0)			-1.5 (-3.2 to 0.2)	-1 (-2.7 to 0.6)	-0.5 (-2.1 to 1.2)
**Running Pain (0–10)**								
**T**_**0**_	5.4 (1.5)	6.1 (1.1)	5.6 (2.1)			-0.2 (-2.1 to 1.7)	0.5 (-1.4 to 2.3)	-0.7 (-2.5 to 1.2)
**T**_**2**_	2.5 (2.4)	2.9 (2.4)	5.1 (2.7)	0.010*	0.10	-2.5 (-5.4 to 0.2)	-2.1 (-4.9 to 0.7)	-0.4 (-3.3 to 2.3)
**T**_**24**_	1.2 (1.3)	1.5 (2.0)	4.5 (1.4)			-3.2 (-5.1 to -1.3)**†**	-2.9 (-4.8 to -1)**†**	-0.3 (-2.2 to 1.5)
**Kujala (0–100)**								
**T**_**0**_	82.7 (6.9)	84.6 (7.8)	76.6 (12.2)			6.1 (-4.4 to 16.6)	8 (-2.5 to 18.5)	-1.9 (-12.4 to 8.6)
**T**_**2**_	86.7 (7.1)	84.9 (8.7)	75.9 (9.7)	0.019*	0.09	10.8 (1.0 to 20.6)**†**	9.0 (-0.8 to 18.8)	1.8 (-7.9 to 11.6)
**T**_**24**_	89.5 (8.1)	84.3 (10.5)	88.6 (5.4)			0.9 (-8.4 to 10.3)	-4.3 (-13.7 to 5.1)	5.2 (-4.1 to 14.6)

T_0_, baseline; T_2_, week 2; T_24_, week 24.

*Significant group x time interaction (p<0.05).

†Significant between-group difference (p<0.05).

### Running biomechanics

A significant group x time interaction and a medium effect size were found for vertical tibial acceleration (p = 0.029, η^2^G = 0.06) and cadence (p = 0.004, η^2^G = 0.07). Both intervention groups decreased vertical tibial acceleration, with a large effect size, compared to the control group at T_24_ (Impact x Control—MD −1.9, 95% CI −3.3 to −0.4, p = 0.009, *g* = -1.28; Cadence x Control—MD −1.6, 95% CI −3.0 to −0.1, p = 0.033, *g* = -1.10). Both intervention groups increased cadence, with a large effect size, compared to the control group at T_24_ (Impact x Control—MD 10, 95% CI 1.3 to 18.5, p = 0.020, *g* = 2.10; Cadence x Control—MD 10.3, 95% CI 1.6 to 18.8, p = 0.016, *g* = 1.09). A significant group x time interaction and a medium effect size were found for ankle dorsiflexion (p = 0.001, η^2^G = 0.08); however, the pairwise comparison did not show a true between-group difference at T_2_ (Impact x Control—MD 2.7, 95% CI −1.7 to 7.1, p = 0.358, *g* = 0.70; Cadence x Control—MD 0.7, 95% CI −3.7 to 5.2, p = 0,960, *g* = 0.19). and T_24_ (Impact x Control—MD −2.1, 95% CI −5.5 to 1.4, p = 0,363, *g* = -0.72; Cadence x Control—MD −3.4, 95% CI −6.8 to 0.1, p = 0,059, *g* = -1.10). No significant group x time interaction and small effect sizes were found for contralateral pelvic drop (p = 0.605, η^2^G = 0.01), hip adduction (p = 0.379, η^2^G = 0.01), knee flexion at midstance (p = 0.264, η^2^G = 0.02), tibial inclination at initial contact (p = 0.616, η^2^G = 0.01), and foot inclination at initial contact (p = 0.699, η^2^G = 0.00) Table 3.

**Table 3:** Mean (SD) and mean difference and 95% CIs for running biomechanics (n = 30).

	Mean (SD)					Between-group difference (95% CI)		
Outcomes	Impact	Cadence	Control	P	η^2^G	Impact x	Cadence x	Impact x
						Control	Control	Cadence
**VTA (g)**								
**T**_**0**_	7.1 (2.4)	6.3 (1.6)	7.0 (1.8)			0.1 (-2.1 to 2.2)	-0.7 (-2.9 to 1.5)	0.8 (-1.4 to 3.0)
**T**_**2**_	6.1 (2.3)	6.9 (1.9)	6.6 (1.6)	0.029*	0.06	-0.5 (-2.7 to 1.7)	0.3 (-1.9 to 2.5)	-0.8 (-3.0 to 1.4)
**T**_**24**_	5.6 (0.9)	5.9 (0.8)	7.5 (1.8)			-1.9 (-3.3 to -0.4)**†**	-1.6 (-3.0 to -0.1)**†**	-0.3 (-1.7 to 1.1)
**Cadence (steps/min)**								
**T**_**0**_	172.4 (9.9)	167.6 (6.4)	168.4 (7.1)			4 (-5.0 to 13.0)	-0.8 (-9.8 to 8.3)	4.8 (-4.2 to 13.8)
**T**_**2**_	173.3 (9.8)	176.8 (7.3)	169.2 (6.6)	0.004*	0.07	4.1 (-5.0 to 13.2)	7.6 (-1.5 to 16.8)	-3.5 (-12.7 to 5.6)
**T**_**24**_	179.0 (2.1)	179.3 (11.1)	169.0 (6.4)			10 (1.3 to 18.5)**†**	10.3 (1.6 to 18.8)**†**	-0.3 (-8.8 to 8.2)
**CPD (°)**								
**T**_**0**_	4.7 (2.8)	5.2 (1.5)	6.4 (3.5)			-1.7 (-4.8 to 1.4)	-1.2 (-4.4 to 1.8)	-0.4 (-3.5 to 2.7)
**T**_**2**_	3.7 (2.0)	4.9 (1.2)	6.6 (2.8)	0.605	0.01	-2.8 (-5.3 to -0.4)	-1.7 (-4.1 to 0.7)	-1.1 (-3.6 to 1.2)
**T**_**24**_	4.8 (3.2)	6.6 (2.1)	7.8 (2.5)			-2.9 (-6 to 0.1)	-1.1 (-4.2 to 1.9)	-1.8 (-4.9 to 1.2)
**Hip adduction (°)**								
**T**_**0**_	75.3 (4.7)	78.4 (3.2)	76.1 (6.4)			-0.8 (-6.5 to 4.8)	2.2 (-3.4 to 7.9)	-3.1 (-8.8 to 2.5)
**T**_**2**_	77.4 (3.9)	78.6 (2.3)	75.9 (5.4)	0.379	0.01	1.5 (-3.1 to 6.2)	2.7 (-1.9 to 7.3)	-1.1 (-5.8 to 3.5)
**T**_**24**_	74.8 (4.5)	76.7 (2.0)	75.3 (6.6)			-0.5 (-5.9 to 4.9)	1.3 (-4.1 to 6.8)	-1.8 (-7.3 to 3.6)
**Knee flexion (°)**								
**T**_**0**_	43.9 (4.6)	42.3 (5.1)	42.3 (4.5)			1.6 (-3.7 to 7.0)	0 (-5.4 to 5.4)	1.6 (-3.7 to 7.0)
**T**_**2**_	44.9 (6.0)	39.5 (4.8)	42.3 (5.1)	0.264	0.02	2.6 (-3.5 to 8.7)	-2.7 (-8.9 to 3.3)	5.4 (-0.7 to 11.5)
**T**_**24**_	42.5 (5.0)	40.8 (5.2)	40.4 (2.7)			2.0 (-3.0 to 7.1)	0.4 (-4.6 to 5.5)	1.6 (-3.4 to 6.7)
**Ankle dorsiflexion (°)**								
**T**_**0**_	27.4 (4.6)	26.7 (3.5)	25 (2.8)			2.4 (-1.8 to 6.6)	1.7 (-2.5 to 5.9)	0.7 (-3.5 to 4.9)
**T**_**2**_	27.8 (3.7)	25.9 (4.3)	25.1 (3.7)	0.001*	0.08	2.7 (-1.7 to 7.1)	0.7 (-3.7 to 5.2)	1.9 (-2.5 to 6.4)
**T**_**24**_	26.8 (3.2)	25.5 (3.5)	28.9 (2.3)			-2.1 (-5.5 to 1.4)	-3.4 (-6.8 to 0.1)	1.3 (-2.1 to 4.8)
**Tibia inclination (°)**								
**T**_**0**_	9.5 (2.6)	7.2 (4.4)	7.7 (2.9)			1.7 (-2.1 to 5.6)	-0.5 (-4.4 to 3.4)	2.2 (-1.6 to 6.1)
**T**_**2**_	10.5 (4.7)	5.9 (3.6)	8.2 (2.9)	0.616	0.01	2.2 (-2.1 to 6.6)	-2.3 (-6.7 to 2.1)	4.5 (0.1 to 9.0)
**T**_**24**_	8.8 (2.8)	6.5 (6.3)	6.8 (3.9)			1.9 (-3.3 to 7.2)	-0.3 (-5.6 to 4.9)	2.3 (-2.9 to 7.5)
**Foot inclination (°)**								
**T**_**0**_	11.4 (4.4)	8.3 (8.6)	11.5 (4.2)			-0.1 (-6.9 to 6.7)	-3.2 (-10.2 to 3.7)	3.1 (-3.8 to 10.1)
**T**_**2**_	11.8 (5.5)	6.1 (6.5)	11.3 (3.6)	0.699	0.00	0.4 (-5.6 to 6.5)	-5.1 (-11.4 to 1.0)	5.6 (-0.5 to 11.9)
**T**_**24**_	9.4 (4.6)	3.0 (11.4)	7.3 (6.0)			2.0 (-6.8 to 10.9)	-4.3 (-13.4 to 4.7)	6.4 (-2.6 to 15.5)

T_0_, baseline; T_2_, week 2; T_24_, week 24. VTA, Vertical Tibial Acceleration. CPD, Contralateral Pelvic Drop. The peaks in degrees of knee flexion and ankle dorsiflexion were evaluated at midstance, while tibial and foot inclination were evaluated at initial contact.

*Significant group x time interaction (p<0.05).

†Significant between-group difference (p<0.05).

## Discussion

To our knowledge, our study is the first that assessed the effects of a feasible program focused on tibial acceleration in runners with PFP, and one of the few RCTs that evaluated the cadence strategy. Both gait retraining programs were more effective in improving running pain six months after the protocol. The gait retraining program on impact was more effective in improving knee function immediately post-training.

Our findings on pain and knee function are in accordance with previous RCTs [13, 14]. A combination of increased cadence and minimalist shoes promoted differences in knee function and worst pain, but not on average pain compared with a prefabricated orthoses group [13]. An increase in cadence with education on load management did not provide additional benefits in pain and symptoms compared with education alone [14]. The differences in tibial acceleration and cadence found only at week twenty-four may be associated with improvements in running pain. It is possible that simple changes from a habitually painful pattern [22] were responsible for the results in knee function found in week two.

No differences in lower limb kinematics were found in the follow-up time points assessed. Our findings corroborate with a two-week program [17] that did not find effects on knee flexion and hip adduction and disagree with two studies that used four [16] and six weeks [15] programs respectively and found moderate/large effects in knee flexion, hip adduction, and contralateral pelvic drop. It can be suggested that two-weeks of using a strategy that does not address lower limbs kinematics directly may not be sufficient to modify these variables in individuals with PFP.

Scientific and clinical implications can be formulated based on the results of this study. Partially supervised programs focused on tibial acceleration or cadence are capable of reducing running pain more so than no intervention at the six-months point. Participants in a supervised protocol focusing on tibial acceleration were unable to transfer the results outside the clinic [40]. These results show the relevance of partially supervised protocols, which allow exposure to outdoor training during retraining. Additionally, our findings are important considering that PFP may not resolve spontaneously and has the potential to become chronic. Pain during functional activities such as running and a longer period of PFP were listed as important factors for a worse prognosis with incomplete recovery at 5 to 8 years [4, 5].

The reductions in running pain occurred regardless of differences in lower limb kinematics. Increasing step rate, adopting a forefoot striking and running softer produced reductions in running-related knee pain and peak PFJ force concurrently [41]. Therefore, differences in running pain may be attributed to reductions in PFJ forces rather than changes in kinematic behavior. Health practitioners may incorporate a decrease in tibial acceleration or an increase in cadence to assist in the management of this condition.

Some limitations of the current study need to be outlined. Non-rearfoot strike runners with a cadence greater than 170 steps/min or tibial acceleration lesser than 8 g were included to promote better recruitment rates. The presence of these characteristics may have influenced the capability of the participants to reach the thresholds established. Participants without important deviations in lower limb kinematics were included. The capability of the strategies chosen to modify lower limb kinematics was limited. The recommendations for biomechanical analysis include the use of a camera with 120 fps [42]. We cannot rule out that our equipment may have influenced the results of the kinematic analysis. A larger sample with a better motion capture system could have provided better insights into changes in running biomechanics. Finally, running volumes were not collected over the 6-months period. Higher mileage or an abrupt increase in running volume could have influenced the primary outcomes.

Future RCTs should investigate the effects of gait retraining programs with a better proportion of supervised and non-supervised sessions or that implement a full non-supervised in-field design in runners with PFP. The use of these programs in a multimodal approach with interventions focusing on pain education, load management, hip/knee strengthening, and psychosocial aspects would be of high relevance in order to promote better recovery rates in this population. Programs based on tibial acceleration [12], cadence [14], and hip adduction [43] reported improvements in pain levels followed by changes in loading rates. However, gait retraining may affect symptoms because of reasons unrelated to biomechanical changes. Therefore, studies to clarify the mechanisms related to the positive effects on clinical outcomes in this population are necessary. The results obtained only reflect this current Brazilian population. The use of data from other populations are necessary for validation.

## Conclusion

Compared to no intervention, two-week partially supervised gait retraining programs focusing on impact and cadence were more effective in improving running pain six months after the protocol in a sample of Brazilian runners with PFP. Additionally, the two-week partially supervised gait retraining program focused on impact was more effective in improving knee function immediately post-training. No differences in usual pain and lower limb kinematics were found in the follow-up time points assessed. As expected, one gait retraining group was not superior to the other.

## Supporting information

S1 Checklist CONSORT 2010 checklist of information to include when reporting a randomised trial*. (DOC)

S1 File Study protocol sent for ethics committee approval. (PDF)

S1 Data (XLSX)
