# Supplementary material for: Effects of two gait retraining programs on pain, function, and lower limb kinematics in runners with patellofemoral pain: A randomized controlled trial
Source: PLoS One. 2024 Jan 10;19(1):e0295645. doi: 10.1371/journal.pone.0295645 (PMC10781021; doi:10.1371/journal.pone.0295645)
Supplement: S1 File — (PDF) [file pone.0295645.s002.pdf]

UNIVERSIDADE DE BRASÍLIA

FACULDADE DE CEILÂNDIA

EFEITOS DE DOIS PROGRAMAS DE RETREINAMENTO DE CORRIDA SOBRE A DOR, FUNÇÃO E CINEMÁTICA DOS MEMBROS INFERIORES DE CORREDORES COM DOR PATELOFEMORAL: ENSAIO CLÍNICO RANDOMIZADO COM FOLLOW-UP DE SEIS MESES

José Roberto de Souza Júnior

Pedro Henrique Reis Rabelo

Thiago Vilela Lemos

João Paulo Chieregato Matheus

Brasília, 2019

UNIVERSIDADE DE BRASÍLIA
FACULDADE DE CEILÂNDIA

EFEITOS DE DOIS PROGRAMAS DE RETREINAMENTO DE CORRIDA SOBRE A DOR, FUNÇÃO E CINEMÁTICA DOS MEMBROS INFERIORES DE CORREDORES COM DOR PATELOFEMORAL: ENSAIO CLÍNICO RANDOMIZADO COM FOLLOW-UP DE SEIS MESES

José Roberto de Souza Júnior
Pedro Henrique Reis Rabelo
Thiago Vilela Lemos
João Paulo Chieregato Matheus

Projeto de pesquisa de doutorado do programa de Pós-graduação em Ciências e Tecnologias em Saúde elaborado para submissão ao Comitê de Ética em Pesquisa para análise e parecer ético

Brasília, 2019

RESUMO

Introdução: A corrida é um esporte que vem ganhando cada vez mais popularidade em todo o mundo, entretanto com o crescente número de adeptos começou a se observar também um alto número de lesões, entre elas a dor patelofemoral. Buscando a prevenção ou o tratamento da dor patelofemoral algumas modalidades de tratamento têm sido utilizadas, dentre estas destaca-se o retreinamento de corrida. **Objetivos:** analisar a influência imediata e em curto prazo de dois programas de retreinamento de corrida sobre a dor, função e cinemática dos membros inferiores de corredores com dor patelofemoral. De forma secundária, investigar se aspectos funcionais e cinemáticos se associam com dor patelofemoral. **Métodos:** Ensaio clínico controlado randomizado (1:1:1), cego, com follow-up de seis meses, que será executado no Instituto Trata, Goiânia. A amostra será composta por corredores de rua com idade entre 18-45 anos que apresentem dor anterior no joelho acima de 3 pontos na Escala Visual Analógica (EVA) durante a corrida e uma tarefa funcional. Inicialmente serão selecionados sujeitos com dor patelofemoral e sem dor patelofemoral e estes serão divididos em grupos A e B respectivamente. Posteriormente os sujeitos do grupo A serão alocados em três grupos: Grupo C (Retreinamento de corrida com foco no impacto); Grupo D (Retreinamento de corrida com foco na cadência); Grupo C (Controle). A dor será avaliada por meio da Escala Visual Analógica da dor, a função por meio da Escala de Desordens Patelofemorais e os aspectos cinemáticos do tronco e membros inferiores por meio de um sistema de análise do movimento bidimensional. Testes especiais e funcionais também serão realizados. O retreinamento será realizado por meio de 8 sessões divididas em 2 semanas com duração de 15 a 30 minutos sendo 2 presenciais e 6 de forma não supervisionada. As avaliações ocorrerão antes, imediatamente e seis meses após o retreinamento de corrida. Os dados serão analisados no SPSS (Statistical Package for Social Sciences) versão 22.0, considerando-se nível de significância de $p<0,05$. **Resultados esperados:** Espera-se que fatores cinemáticos e funcionais se associem com dor patelofemoral e que os protocolos de retreinamento propostos modifiquem a dor, função e os aspectos cinemáticos de corredores com dor patelofemoral e que tais resultados sejam mantidos por seis meses.

1. INTRODUÇÃO

A corrida é frequentemente requisitada como um exercício para condicionamento, lazer e competição e tem apresentado um número crescente de adeptos em todo o mundo. A incidência de lesões nessa população é alta e pode variar de 19 a 92% de acordo com a definição escolhida para conceituar lesão nesses sujeitos (SARAGIOTTO et al., 2014; YAMATO et al., 2015).

As lesões esportivas apresentam alta complexidade e são o resultado da conexão multidirecional de diferentes fatores, por meio do entendimento da interação destes pode-se estabelecer um perfil de risco e dessa forma reduzir as chances de um atleta vir a apresentar uma lesão (BITTENCOURT et al., 2016).

Em relação à avaliação, atualmente as pesquisas estão voltadas principalmente para as interações entre os aspectos cinemáticos (queda da pelve; adução da coxa; rotação interna do quadril; ângulo do joelho; ângulo do tornozelo) e cinéticos (pico de impacto vertical; pico de aceleração da tíbia; taxa de impacto vertical média; taxa de impacto vertical instantânea) já que estes apresentam relação com as principais lesões presentes em corredores (CHEUNG; DAVIS, 2011; CLANSEY et al, 2014; CROWELL; DAVIS, 2011; CROWELL; MILNER; HAMILL, 2010; DIEBAL; GREGORY; ALITZ, 2011; DIEBAL et al, 2012; ERIKSSON; HALVORSEN; GULLSTRAND, 2011; MESSIER; CIRILLO, 1989; NOEHREN; SCHOLZ; DAVIS, 2011; WILLY; SCHOLZ; DAVIS, 2012). Em relação ao tratamento, uma intervenção que têm sido utilizada nos últimos anos e que tem apresentado resultados positivos não só na prevenção como também no controle dos sintomas gerados por tais lesões é o retreinamento de corrida (DIEBAL et al., 2012; ERIKSSON; HALVORSEN; GULLSTRAND, 2011; NOEHREN et al., 2009; TATE; MILNER, 2017; WILLY; SCHOLZ; DAVIS, 2012).

O retreinamento de corrida é uma modalidade de tratamento que visa modificar os padrões biomecânicos que podem estar associados às lesões em corredores (AGRESTA; BROWN, 2015). Alguns estudos já foram realizados avaliando os efeitos do retreinamento de corrida em corredores com dor patelofemoral, as estratégias mais usuais demandam feedback auditivo e/ou visual (LEIBBRANDT; LOW, 2017; NOEHREN; SCHOLZ; DAVIS, 2011; WILLY; SCHOLZ; DAVIS, 2012), mudanças na cadência (BONNACI et al, 2017; DOS SANTOS et al., 2019; ESCULIER et al., 2017) e no padrão de contato

inicial com o solo (DOS SANTOS et al., 2019; ROPER et al., 2016), para modular os aspectos cinemáticos e cinéticos previamente avaliados.

Tais estudos encontraram que os programas de retreinamento de corrida tiveram efeitos positivos sobre a dor (BONNACI et al., 2017; DOS SANTOS et al., 2019; ESCULIER et al., 2017; LEIBBRANDT; LOW, 2017; NOEHREN; SCHOLZ; DAVIS, 2011; ROPER et al., 2016; WILLY; SCHOLZ; DAVIS, 2012), função (DOS SANTOS et al., 2019; ESCULIER et al., 2017; LEIBBRANDT; LOW, 2017; NOEHREN; SCHOLZ; DAVIS, 2011; WILLY; SCHOLZ; DAVIS, 2012) e biomecânica dos corredores com dor patelofemoral (ESCULIER et al., 2017; LEIBBRANDT; LOW, 2017; NOEHREN; SCHOLZ; DAVIS, 2011; ROPER et al., 2016; WILLY; SCHOLZ; DAVIS, 2012) e que tais resultados ainda foram mantidos por 1 (NOEHREN; SCHOLZ; DAVIS, 2011; ROPER et al., 2016; WILLY; SCHOLZ; DAVIS, 2012), 3 (WILLY; SCHOLZ; DAVIS, 2012) e aproximadamente 6 meses (DOS SANTOS et al., 2019; ESCULIER et al., 2017).

Dos estudos realizados com retreinamento de corrida, somente um apresentou grupo controle no qual nenhuma intervenção foi realizada (ROPER et al., 2016) e somente dois acompanharam os resultados por um período de 6 meses (DOS SANTOS et al., 2019; ESCULIER et al., 2017). Os programas foram consistidos de 8 sessões durante 2 semanas (DOS SANTOS et al., 2019; LEIBBRANDT; LOW, 2017; NOEHREN; SCHOLZ; DAVIS, 2011; ROPER et al., 2016; WILLY; SCHOLZ; DAVIS, 2012) ou de sessões durante 6 (BONNACI et al., 2017) e 8 semanas (ESCULIER et al., 2017).

Nesse sentido, tem-se a necessidade de ensaios clínicos que avaliem a eficácia de programas de retreinamento de corrida, que tenham um período de follow-up e que proponham um modelo de retreinamento mais próximo da prática clínica, visto que tais programas são onerosos e demandam tempo por parte do participante e do terapeuta. Estudos com dor patelofemoral são necessários visto que 40% dos indivíduos tratados tem recorrência dos sintomas dentro de 1 ano e 50% não se recuperam completamente dentro de 5 e 8 anos (COLLINS et al., 2013; LANKHORST et al., 2016).

O objetivo do estudo é verificar os efeitos de dois programas de retreinamento de corrida parcialmente supervisionados sobre a dor, função e cinemática dos membros inferiores de corredores com dor patelofemoral. De forma secundária, investigar a interação

de diferentes aspectos cinemáticos e funcionais, com a presença de dor patelofemoral em corredores.

2. OBJETIVOS

2.1. Objetivo geral

Verificar os efeitos de dois programas de retreinamento de corrida parcialmente supervisionados sobre a dor, função e cinemática dos membros inferiores de corredores com dor patelofemoral.

2.2. Objetivos específicos

Avaliar o conhecimento referente ao retreinamento de corrida dos corredores da cidade de Goiânia.

Investigar se aspectos cinemáticos e funcionais apresentam associação com dor patelofemoral durante a corrida em corredores.

Investigar se aspectos funcionais apresentam associação com aspectos cinemáticos durante a corrida em sujeitos com dor patelofemoral.

3. MATERIAIS E MÉTODOS

3.1. Tipo de estudo

Inicialmente será realizado um estudo transversal para verificar o conhecimento dos corredores de Goiânia sobre o retreinamento de corrida e para avaliar a interação entre aspectos cinemáticos e funcionais com a presença de dor patelofemoral. Tal análise precede a realização do ensaio clínico que visa avaliar a influência de diferentes programas de retreinamento de corrida nestes aspectos. Após a avaliação inicial do conhecimento dos corredores sobre retreinamento de corrida, os sujeitos de acordo com os critérios de inclusão e exclusão explicitados abaixo serão selecionados e incluídos nos grupos A e B. O grupo A será de sujeitos com dor patelofemoral, enquanto que o grupo B será de sujeitos sem dor patelofemoral. Para o ensaio clínico, serão incluídos somente os sujeitos do grupo A (com dor patelofemoral), estes serão divididos em três subgrupos chamados de grupo C (Retreinamento de corrida com foco no impacto), grupo D (Retreinamento de corrida com foco na cadência) e controle. Este segundo momento da pesquisa será um Ensaio Clínico Controlado Randomizado (1:1:1), cego, com follow-up de 6 meses, registrado no REBEC (Registro Brasileiro de Ensaios Clínicos) e realizado de acordo com as recomendações do CONSORT (Consolidated Standards of Reporting Trials) (SCHULZ et al, 2010). O estudo será submetido para apreciação no Comitê de Ética em Pesquisa (CEP). O tamanho da amostra foi calculado considerando-se o ensaio clínico. O cálculo amostral foi realizado no software G*Power, versão 3.1, por meio de uma análise pareada, considerando-se alfa de 95%, Power de 95%, tamanho do efeito de 1.51 e a variável dor (FAUL et al, 2009). A amostra determinada foi de 7 sujeitos, considerando-se 10% de perca amostra, a amostra final será de 8 sujeitos em cada grupo (C x D x Controle). O cálculo amostral foi realizado de acordo com o estudo de Neal et al., (2018) que avaliou a viabilidade de uma programa de retreinamento de corrida com foco na cadência e encontrou uma diferença de 2.1 pontos na Escala Visual Analógica entre a dor média antes e após o programa e tamanho de efeito (*d* de Cohen) de 1.7. Além dos 24 sujeitos com dor patelofemoral (8 em cada grupo), outros 24 sujeitos sem dor patelofemoral pareados de acordo com características antropométricas serão recrutados. O recrutamento do estudo ocorrerá após aprovação do Comitê de Ética em

Pesquisa (CEP) e obtenção do registro de ensaio clínico por meio do Registro Brasileiro de Ensaios Clínicos (REBEC).

3.2. Local

Este estudo será realizado no Instituto Trata situado na Rua T-53, 1043 – Setor Bueno, Goiânia, Goiás, CEP: 74215-150

3.3 Critérios de Inclusão

Para a análise transversal serão incluídos participantes que apresentam dor patelofemoral e que não apresentam dor patelofemoral. Os critérios de inclusão para quem tem dor patelofemoral serão: sujeitos de ambos os sexos, corredores de rua de retropé com cadência menor que 170 passos por minuto, com idade entre 18 e 45 anos, que apresentem dor anterior no joelho acima de 3 pontos na Escala Visual Analógica (EVA) durante a corrida e em pelo menos uma atividade entre agachar, subir e descer degraus, ajoelhar e estender o joelho de forma resistida, e que treinem/corram com velocidade média entre 10-12 Km/hora. Os critérios de inclusão para quem não tem dor patelofemoral serão: corredores de rua com os mesmos critérios de gênero e idade do grupo anterior e sem qualquer sintoma relacionado à dor patelofemoral.

3.4. Critérios de Exclusão

Os sujeitos com dor patelofemoral serão excluídos do ensaio clínico, caso apresentem outras doenças nos membros inferiores, antecedentes cirúrgicos no último ano, e que não tenham interesse para realizar um programa de retreinamento de 2 semanas.

3.5. Randomização

Em relação ao ensaio clínico, os participantes com dor patelofemoral serão informados que eles poderão ser alocados em um dos três grupos do estudo e dessa forma "participar do protocolo de retreinamento com foco no impacto", "participar do protocolo de retreinamento com foco na cadência" ou "não participar do protocolo de retreinamento de corrida".

Uma sequência simples de randomização será gerada no software Microsoft Excel por um dos investigadores do estudo que não estará diretamente envolvido nas avaliações ou tratamento. Após as avaliações iniciais o terapeuta irá abrir o envelope de randomização e os participantes serão alocados em um dos três grupos do estudo: Grupo C (Retreinamento de corrida com foco no impacto); Grupo D (Retreinamento de corrida com foco na cadência); Controle. A alocação será oculta por meio de envelopes opacos, lacrados e numerados consecutivamente. Os protocolos de retreinamento serão realizados imediatamente após a alocação.

3.6. Cegamento

Em relação ao ensaio clínico, o avaliador dos desfechos e o investigador que fará as análises estatísticas dos dados serão cegos em relação ao grupo de alocação dos participantes do estudo. Devido à natureza das intervenções, não será possível que o terapeuta e os participantes sejam cegos quanto as condições de tratamento.

3.7. Follow-up

As avaliações serão feitas antes da randomização, imediatamente após os protocolos de retreinamento de corrida e seis meses após o término das intervenções do estudo. Todas as avaliações serão conduzidas pessoalmente. Entre o término do protocolo e a avaliação seis meses após este serão enviadas mensagens mensais questionando o participante sobre frequência, volume e intensidade de treino além de desconfortos durante os treinamentos.

3.8 Instrumentos

Os seguintes instrumentos serão utilizados para a coleta dos dados:

- **Termo de Consentimento Livre e Esclarecido:** contém informações de forma simples e resumida, sobre o título, objetivos, metodologia, benefícios e riscos do estudo, sigilo dos dados e imagens, da participação voluntária do sujeito e da garantia de que este pode desistir do estudo a qualquer momento.

- **Questionário epidemiológico:** será utilizado para caracterizar a amostra e avaliar o conhecimento sobre o retreinamento de corrida. Contém as seguintes informações: nome,

idade (anos), massa corporal (quilogramas), estatura (metros), Índice de Massa Corporal (quilogramas por metro quadrado), antecedentes patológicos e cirúrgicos, características da corrida (tempo de prática do esporte, velocidade/pace, volume, frequência, provas/ano) e perguntas referentes ao retreinamento de corrida.

- **Escala Visual Analógica da Dor (EVA):** será utilizada para mensurar a intensidade da dor. Consiste de uma escala numérica de 0 a 10 pontos, onde 0 significa ausência de dor e 10 significa o máximo de dor já vivenciada pelo sujeito (PIMENTA, 1994).

- Escala de Desordens Patelofemorais (ANEXO 1): será utilizada para avaliar a função. Consiste de um questionário traduzido e validado para a língua portuguesa que contém 13 questões que avaliam a severidade dos sintomas e a limitação em diferentes atividades relacionadas à dor patelofemoral. Apresenta uma pontuação entre 0 a 100 onde quanto menor a pontuação pior a função (AQUINO et al, 2011).

- **Sistema de Análise do Movimento:** será utilizado para avaliar a cinemática dos membros inferiores. A avaliação ocorrerá por meio de vídeos digitais usando duas webcams (MyoVideo 139 HD Color Webcam) de amostragem a 30 quadros por segundo e dois leds (LED Floodlight). Marcadores reflexivos (19 mm) serão colocados no manúbrio do esterno e bilateralmente na Espinha Ilíaca Ântero-superior, trocânter maior, epicôndilo lateral do fêmur, cabeça da fíbula e maléolo lateral (DINGENEN et al., 2018a). Todos os participantes serão instruídos a correr em 10-12 km / hora em uma esteira motorizada (Movement XL 1600). Um período de aclimatação de 6 minutos na esteira será utilizado antes da avaliação da cinemática da corrida (LAVCANSKA; TAYLOR; SCHACHE, 2005). A câmera do plano frontal será colocada em um tripé portátil perpendicular ao plano frontal, a uma altura de 1,05m e a uma distância de 2,0m da esteira (DINGENEN et al., 2018a). A câmera do plano sagital será colocada em um tripé portátil, perpendicular ao plano sagital, a uma altura de 0,80m e a uma distância de 1,40m da esteira (DINGENEN et al., 2018a). Os vídeos serão analisados usando o software MyoResearch 3.14 - MyoVideo (Noraxon U.S.A. Inc.). No plano frontal, os ângulos avaliados serão: queda pélvica contralateral; adução do quadril (ângulos avaliados durante o apoio médio) (DINGENEN et al., 2018a; DINGENEN et al., 2018b; MAYKUT et al., 2015). No plano sagital, os ângulos avaliados serão: inclinação do pé; inclinação da tíbia; dorsiflexão do tornozelo; flexão do joelho (dois primeiros serão avaliados durante o contato inicial e os dois últimos no apoio

médio) (DINGENEN et al., 2018a; PIPKIN et al., 2016; SOUZA, 2016). No plano frontal, a posição de aterrissagem mais profunda será determinada visualmente, avançando lentamente o vídeo frame por frame (DINGENEN et al., 2018a; DINGENEN et al., 2018b; MAYKUT et al., 2015). O apoio médio no plano sagital será definido visualmente da mesma forma que no plano frontal e é tipicamente o ponto em que a perna em balanço cruza a perna de apoio (DINGENEN et al., 2018b). O contato inicial será determinado visualmente, avançando lentamente o vídeo, frame por frame, e será definido como a primeira vez que o pé tocou o chão (PIPKIN et al., 2016). Para analisar os ângulos propostos, serão considerados sete passos (DINGENEN et al., 2018a).

- **PHAST (Physiotherapy Assessment Tool):** será utilizado para avaliar os aspectos funcionais (rigidez passiva dos rotadores laterais do quadril, força isométrica dos rotadores laterais do quadril, força isométrica dos abdutores do quadril e amplitude de movimento de dorsiflexão do tornozelo em cadeia cinética fechada). É um aplicativo que reúne diversos testes com objetivos específicos. Os aspectos avaliados e a forma de avaliação estão descritas a seguir:

Rigidez dos rotadores laterais do quadril: O participante é posicionado em decúbito ventral com o joelho flexionado a 90°, o pesquisador movimenta passivamente o membro inferior a ser testado em rotação medial do quadril. Com um inclinômetro posicionado 5 centímetros abaixo da tuberosidade anterior da tíbia será feito o registro em graus da amplitude de rotação medial do quadril (CARVALHAIS et al, 2011).

Dinamometria isométrica dos abdutores do quadril: O participante é posicionado em decúbito lateral em uma maca com os membros superiores posicionados na frente do corpo. Uma faixa estabilizadora é utilizada para fixação do tronco e outra, posicionada cinco centímetros superiormente a interlinha articular do joelho, utilizada para limitar a amplitude de movimento de abdução de quadril. O participante irá realizar uma abdução isométrica máxima com o membro inferior testado durante cinco segundos. Este procedimento será realizado três vezes com intervalo de quinze segundos entre cada contração isométrica. Durante o teste, será dado incentivo verbal para garantir que o participante realize a contração máxima. Registra-se a função muscular dos abdutores do quadril em Nm/Kg por meio de um dinamômetro manual (Hand Held –Microfet2 ®) posicionado cinco centímetros acima da interlinha articular do joelho.

Dinamometria isométrica dos rotadores laterais do quadril: O participante é posicionado em sedestação, em uma maca com os membros superiores cruzados na altura do toráx. Uma faixa estabilizadora é utilizada para fixação da pelve com objetivo de minimizar os movimentos do tronco. O participante irá realizar rotação lateral isométrica do quadril máxima com o membro inferior testado durante cinco segundos. Este procedimento será realizado três vezes com intervalo de quinze segundos entre cada contração isométrica. Durante o teste, será dado incentivo verbal para garantir que o participante realize a contração máxima. Registra-se a função muscular dos rotadores laterais do quadril em Nm/Kg por meio de um dinamômetro manual (Hand Held – Microfet2 ®) posicionado cinco centímetros acima do maléolo medial do membro a ser avaliado.

Lunge teste: O participante é posicionado de pé, tronco alinhado para uma parede com o membro inferior a ser testado à frente. Sem elevar o calcâneo do membro inferior posterior, o mesmo deve flexionar ativamente o joelho do membro inferior anterior até encosta-lo na parede. Com um inclinômetro posicionado 15 centímetros abaixo da tuberosidade anterior da tíbia será feito o registro em graus da amplitude de dorsiflexão do tornozelo em CCF (BENNELL et al, 1998).

3.9. Protocolo

Grupo C (Retreinamento de corrida com foco no impacto): o protocolo de retreinamento de corrida será realizado quatro vezes por semana, com duração gradativa de 15 para 30 minutos, durante duas semanas, sendo duas sessões de forma supervisionada e as outras seis de forma não supervisionada, as sessões supervisionadas serão a primeira e a quinta sessão. Durante as sessões com supervisão, os participantes irão correr calçados (tênis que utilizam habitualmente) em uma velocidade de 10-12 km/hora (velocidade de treino/confortável) e receberão um feedback visual (aceleração da tíbia captada por meio de um acelerômetro - Tgforce v2.0.0.10) e verbal (comandos dados pelos pesquisadores) durante todo o período da intervenção. O acelerômetro será colocado no aspecto ânteromedial da região distal da tíbia (CROWEL; DAVIS, 2011). Uma televisão posicionada em frente da esteira irá mostrar um gráfico da aceleração da tíbia em tempo real captado pelo acelerômetro. Na tela o participante irá ver uma linha que representa

aproximadamente 50% da média do pico de aceleração da tíbia obtido durante o último minuto de corrida (CROWEL et al., 2010; CROWEL; DAVIS, 2011). O examinador irá orientar o participante a correr mais suave, tornar os passos mais silenciosos e manter o pico de aceleração da tíbia abaixo da linha mostrada na tela (CREABY; SMITH, 2015; CROWEL et al., 2010; CROWEL; DAVIS, 2011). Estas instruções serão dadas durante o primeiro minuto do retreinamento e reforçadas a cada 30 segundos de acordo com a necessidade observada pelo examinador (CREABY; SMITH, 2015). Os participantes serão orientados a manter o novo padrão de corrida durante as demais sessões não supervisionadas realizadas em local de sua preferência (CHAN et al., 2018).

Grupo D (Retreinamento de corrida com foco na cadência): o protocolo de retreinamento de corrida será realizado quatro vezes por semana, com duração gradativa de 15 para 30 minutos, durante duas semanas, sendo duas sessões de forma supervisionada e as outras seis de forma não supervisionada, as sessões supervisionadas serão a primeira e a quinta sessão. Durante as sessões com supervisão, os participantes irão correr calçados (tênis que utilizam habitualmente) em uma velocidade de 10-12 km/hora (velocidade de treino/confortável), receberão orientações com relação a sua cadência e irão correr com o auxílio de um metrônomo com uma cadência ajustada entre 7.5 a 10%. Durante as demais sessões, os participantes terão que realizar o retreinamento de corrida de forma idêntica ao realizado na sessão supervisionada em local de sua preferência.

Para garantir que os participantes dos grupos C e D realizem os protocolos e da forma como estes devem ser realizados foi realizada inicialmente a sessão supervisionada. Outras medidas adotadas pelos pesquisadores são: (I) orientação após a sessão supervisionada sobre a forma como as sessões não supervisionadas devem ocorrer; (II) entrega de uma tabela mostrando como as sessões não supervisionadas devem ocorrer (veja tabela 1); (III): mensagem de texto diária no telefone dos participantes lembrando os mesmos de realizarem as sessões como previamente orientado.

Tabela 1. Protocolo que será realizado pelos participantes do estudo

Semana	Dia	Tipo de treino	Tempo de feedback	Tempo de treino
1	1	Supervisionado	15 minutos	15 minutos
	2	Não supervisionado	18 minutos	18 minutos
	3	Não supervisionado	21 minutos	21 minutos

	4	Não supervisionado	24 minutos	24 minutos
2	5	Supervisionado	21 minutos	27 minutos
	6	Não supervisionado	15 minutos	30 minutos
	7	Não supervisionado	9 minutos	30 minutos
	8	Não supervisionado	3 minutos	30 minutos

Fonte: Próprios autores.

Grupo Controle: não receberá quaisquer estratégias de retreinamento de corrida ou orientações até o término do estudo. Após essa etapa os participantes receberão orientações pertinentes a sua condição baseadas nas avaliações biomecânicas previamente realizadas.

3.10. Procedimentos

Inicialmente os pesquisadores divulgarão o estudo por meio de panfletos em assessorias de corrida de Goiânia, mídias sociais e em eventos de corrida realizados na cidade de Goiânia. Durante o primeiro contato, os objetivos e procedimentos do estudo serão esclarecidos e aqueles que concordarem e assinarem o TCLE irão responder o questionário epidemiológico. Aqueles que se encaixarem nos critérios de inclusão e exclusão serão distribuídos nos grupos da análise transversal.

Após essa etapa os sujeitos serão convidados para o Instituto Trata onde serão realizadas as avaliações de dor, função, cinemática dos membros inferiores e testes funcionais. Após a realização de tais avaliações, os sujeitos do grupo A serão randomizados nos subgrupos C, D e controle. Nos participantes dos grupos C e D ocorrerá o agendamento das atividades de retreinamento de corrida que ocorrerão nas duas semanas subsequentes as avaliações iniciais. Os corredores alocados no Grupo controle seguirão nas duas semanas subsequentes realizando suas atividades de treino sem interferência dos pesquisadores. Após esse período serão realizadas novamente as avaliações de dor, função, cinemática dos membros inferiores e testes funcionais.

Os sujeitos que realizaram as avaliações e completaram o protocolo de retreinamento de corrida serão acompanhados por um período de seis meses, ao fim deste uma nova reavaliação dos aspectos supracitados será realizada a fim de se observar os efeitos de curto prazo do protocolo utilizado no estudo.

3.11. Análise Estatística

A análise dos dados será realizada no SPSS (Statistical Package for Social Sciences), versão 23.0. Inicialmente será realizada uma análise descritiva com cálculo de média, desvio padrão e intervalo de confiança de 95% para as variáveis quantitativas e frequência e porcentagem para as variáveis qualitativas.

Para avaliar se os aspectos cinemáticos e funcionais se associam com dor patelofemoral será realizada uma regressão linear considerando a presença de dor patelofemoral como variável dependente e os aspectos cinemáticos e funcionais como variáveis independentes. Ainda será realizada uma regressão linear considerando os aspectos cinemáticos como variáveis dependentes e os aspectos funcionais como variáveis independentes, para que sejam respondidos todos os objetivos específicos do projeto.

Para verificar a normalidade dos dados será utilizado o teste de Shapiro-Wilk. Para comparar as variáveis antes, imediatamente e seis meses após o programa de retreinamento de corrida nos grupos do estudo será utilizado a ANOVA de medidas repetidas (dados paramétricos) ou teste de Friedman (dados não paramétricos). Para comparar as variáveis entre os diferentes grupos do estudo será utilizado a ANOVA (dados paramétricos) ou Kruskall-Wallis (dados não paramétricos). Para realizar as comparações entre os pares será utilizado o pos-hoc de Tukey (dados paramétricos) ou teste de Wilcoxon/Mann Whitney U com correção de Bonferroni. Será utilizado nível de significância de $p<0,05$. Será realizada uma análise por intenção de tratar onde os sujeitos serão analisados independentemente da conclusão do protocolo. Caso haja algum participante que não conclua o mesmo, os motivos serão descritos na seção dos resultados do manuscrito.

REFERÊNCIAS BIBLIOGRÁFICAS

AGRESTA, C.; BROWN, A. Gait retraining for injured and healthy runners using augmented feedback: a systematic literature review. **Journal of Orthopaedic & Sports Physical Therapy**, v. 45, n. 8, p. 576-584, 2015.

AQUINO, V. et al. Tradução e adaptação cultural para a língua portuguesa do questionário Scoring of Patellofemoral Disorders: estudo preliminar. **Acta Ortopédica Brasileira**, v. 19, n. 5, 2011.

BENNELL, K et al. Intra-rater and inter-rater reliability of a weight-bearing lunge measure of ankle dorsiflexion. **Australian Journal of Physiotherapy,** v. 44, n. 3, p. 175-180, 1998.

BITTENCOURT, N. F. N. et al. Complex systems approach for sports injuries: moving from risk factor identification to injury pattern recognition—narrative review and new concept. **British Journal of Sports Medicine**, v. 50, n. 21, p. 1309-1314, 2016.

BONACCI, J. et al. Gait retraining versus foot orthoses for patellofemoral pain: a pilot randomised clinical trial. **Journal of science and medicine in sport**, v. 21, n. 5, p. 457-461, 2018.

CARVALHAIS, V. O.; DE ARAÚJO, V. L.; SOUZA, T. R et al. Validity and reliability of clinical tests for assessing hip passive stiffness. **Manual Therapy,** v. 16, n. 3, p.240-245, 2011.

CHAN, Z. Y. S et al. Gait retraining for the reduction of injury occurrence in novice distance runners: 1-year follow-up of a randomized controlled trial. **The American journal of sports medicine**, v. 46, n. 2, p. 388-395, 2018.

CHEUNG, R. T.; DAVIS, I. S. Landing pattern modification to improve patellofemoral pain in runners: a case series. **Journal Orthopaedic and Sports Physical Therapy**, v. 41, p. 914-919, 2011.

CLANSEY, A. C.; HANLON, M.; WALLACE, E. S et al. Influence of tibial shock feedback training on impact loading and running economy. **Medicine Science and Sports Exercise**, v. 46, p. 973-981, 2014.

COLLINS, N. J. et al. Prognostic factors for patellofemoral pain: a multicentre observational analysis. **British Journal of Sports Medicine,** v. 47, n. 4, p. 227-233, 2013.

CREABY, M. W.; SMITH, M. M. Franettovich. Retraining running gait to reduce tibial loads with clinician or accelerometry guided feedback**. Journal of science and medicine in sport**, v. 19, n. 4, p. 288-292, 2016.

CROWELL, H. P.; DAVIS, I. S. Gait retraining to reduce lower extremity loading in runners. **Clinical Biomechanics (Bristol, Avon),** v. 26, p. 78-83, 2011

CROWELL, H. P.; MILNER, C. E.; HAMILL, J et al. Reducing impact loading during running with the use of real-time visual feedback. **Journal of Orthopaedic Sports Physical Therapy,** v. 40, p. 206-213, 2010.

DIEBAL, A. R.; GREGORY, R.; ALITZ, C. Effects of forefoot running on chronic exertional compartment syndrome: a case series. **International Journal of Sports Physical Therapy**, v. 6, p. 312-321, 2011.

DIEBAL, A. R.; GREGORY, R.; ALITZ, C et al. Forefoot running improves pain and disability associated with chronic exertional compartment syndrome. **American Journal of Sports Medicine,** v. 40, p. 1060-1067, 2012.

DINGENEN, B. et al. Test-retest reliability of two-dimensional video analysis during running. **Physical Therapy in Sport**, v. 33, p. 40-47, 2018a.

DINGENEN, B. et al. Are two-dimensional measured frontal plane angles related to three-dimensional measured kinematic profiles during running? **Physical Therapy in Sport**, v. 29, p. 84-92, 2018b.

DOS SANTOS, A. F. et al. Effects of three gait retraining techniques in runners with patellofemoral pain. **Physical Therapy in Sport**, v. 36, p. 92-100, 2019.

ERIKSSON, M.; HALVORSEN, K.A.; GULLSTRAND, L. Immediate effect of visual and auditory feedback to control the running mechanics of well-trained athletes. **Journal of Sports Science**, v. 29, p. 253-262, 2011.

ESCULIER, Jean-Francois. et al. Is combining gait retraining or an exercise programme with education better than education alone in treating runners with patellofemoral pain? A randomised clinical trial. **British Journal of Sports Medicine**, v. 52, n. 10, p. 659-666, 2018.

HEIDERSCHEIT, B. Always on the run. **Journal of Orthopaedic and Sports Physical Therapy**, v. 44, n. 10, 2014.

HESPANHOL JUNIOR, L. C et al. Health and economic burden of running-related injuries in runners training for an event: A prospective cohort study. **Scandinavian journal of medicine & science in sports,** v. 26, n. 9, p. 1091-1099, 2016.

FAUL, F.; ERDFELDER, E.; BUCHNER, A et al. Statistical power analyses using G*Power 3.1: Tests for correlation and regression analyses. **Behavior Research Methods**, v. 41, n. 4, p. 1149-1160, 2009.

LANKHORST, N. E. et al. Factors that predict a poor outcome 5–8 years after the diagnosis of patellofemoral pain: a multicentre observational analysis. **British Journal of Sports Medicine**, v. 50, n. 14, p. 881-886, 2016.

LAVCANSKA, V.; TAYLOR, N. F.; SCHACHE, A. G. Familiarization to treadmill running in young unimpaired adults. **Human movement science**, v. 24, n. 4, p. 544-557, 2005.

LEIBBRANDT, D. C.; LOUW, Q. A. Targeted Functional movement retraining to improve pain, function, and biomechanics in subjects with anterior knee pain: A case series. **Journal of sport rehabilitation,** v. 27, n. 3, p. 218-223, 2018.

MAYKUT, J. N. et al. Concurrent validity and reliability of 2d kinematic analysis of frontal plane motion during running. **International journal of sports physical therapy**, v. 10, n. 2, p. 136, 2015.

MESSIER, S. P.; CIRILLO, K. J. Effects of a verbal and visual feedback system on running technique, perceived exertion and running economy in female novice runners. **Journal of Sports Science, v**. 7, p. 113-126, 1989.

NEAL, B. S. et al. The effects & mechanisms of increasing running step rate: A feasibility study in a mixed-sex group of runners with patellofemoral pain. **Physical Therapy in Sport**, v. 32, p. 244-251, 2018.

NOEHREN, B. W.; DAVIS, I. Can Gait Retraining Change Hip Kinematics And Reduce Symptoms In Runners With Patellofemoral Pain Syndrome? 604May 27 2: 15 PM-2: 30 PM. **Medicine & Science in Sports & Exercise**, v. 41, n. 5, p. 26, 2009.

NOEHREN, B.; SCHOLZ, J.; DAVIS, I. The effect of realtime gait retraining on hip kinematics, pain and function in subjects with patellofemoral pain syndrome. **British Journal of Sports Medicine**, v. 45, p. 691-696, 2011.

PIMENTA, C. A. M. **Escalas de avaliação de dor**. In: TEIXEIRA, M. D (ed.) Dor conceitos gerais. São Paulo: Limay, 1994.

PIPKIN, A. et al. Reliability of a qualitative video analysis for running. **Journal of orthopaedic & sports physical therapy**, v. 46, n. 7, p. 556-561, 2016.

ROPER, J. L. et al. The effects of gait retraining in runners with patellofemoral pain: A randomized trial. **Clinical biomechanics,** v. 35, p. 14-22, 2016.

SARAGIOTTO, B. I et al. What are the main risk factors for running-related injuries? **Sports medicine**, v. 44, n. 8, p. 1153-1163, 2014.

SCHULZ, K. F.; ALTMAN, D. G.; MOHER, D.; FOR THE CONSORT GROUP. CONSORT 2010 Statement: updated guidelines for reporting parallel group randomised trials. **Annals of Internal Medicine,** 2010;152.

SOUZA, R. B. An evidence-based videotaped running biomechanics analysis. **Physical medicine and rehabilitation clinics**, v. 27, n. 1, p. 217-236, 2016.

TATE, J. J.; MILNER, C. E. Sound-intensity feedback during running reduces loading rates and impact peak. **Journal of Orthopaedic & Sports Physical Therapy**, v. 47, n. 8, p. 565-569, 2017.

WILLY, R. W.; SCHOLZ, J.P.; DAVIS, I. S. Mirror gait retraining for the treatment of patellofemoral pain in female runners. **Clinical Biomechanics (Bristol, Avon)**, v. 27, p. 1045-1051, 2012.

YAMATO, T. P.; SARAGIOTTO, B. T.; LOPES, A. D. A consensus definition of running-related injury in recreational runners: a modified Delphi approach. **Journal of Orthopaedic & Sports Physical Therapy**, v. 45, n. 5, p. 375-380, 2015.

UNIVERSITY OF BRASILIA

CEILANDIA FACULTY

EFFECTS OF TWO GAIT RETRAINING PROGRAMS ON PAIN, FUNCTION AND KINEMATICS OF THE LOWER LIMBS OF RUNNERS WITH PATELLOFEMORAL PAIN: A SIX-MONTH FOLLOW-UP RANDOMIZED CLINICAL TRIAL

Jose Roberto de Souza Junior

Pedro Henrique Reis Rabelo

Thiago Vilela Lemos

Joao Paulo Chieregato Matheus

Brasilia, 2019

UNIVERSITY OF BRASILIA

CEILANDIA FACULTY

EFFECTS OF TWO GAIT RETRAINING PROGRAMS ON PAIN, FUNCTION AND KINEMATICS OF THE LOWER LIMBS OF RUNNERS WITH PATELLOFEMORAL PAIN: A SIX-MONTH FOLLOW-UP RANDOMIZED CLINICAL TRIAL

Jose Roberto de Souza Junior

Pedro Henrique Reis Rabelo

Thiago Vilela Lemos

Joao Paulo Chieregato Matheus

Doctoral research project of the Graduate Program in Health Sciences and Technologies prepared for submission to the Research Ethics Committee for analysis and ethical opinion

Brasilia, 2019

ABSTRACT

Introduction: Running is a sport that has been gaining more and more popularity all over the world, however with the growing number of fans, a high number of injuries has also begun to be observed, including patellofemoral pain. Seeking the prevention or treatment of patellofemoral pain, some treatment modalities have been used, among which running retraining stands out. **Objectives:** to analyze the immediate and short-term influence of two gait retraining programs on pain, function and kinematics of lower limbs in runners with patellofemoral pain. Secondarily, to investigate whether functional and kinematic aspects are associated with patellofemoral pain. **Methods:** Randomized controlled clinical trial (1:1:1), blinded, with a follow-up of six months, which will be carried out at Instituto Trata, Goiânia. The sample will consist of street runners aged between 18-45 years old who have anterior knee pain above 3 points on the Visual Analogue Scale (VAS) during running and a functional task. Initially, subjects with patellofemoral pain and without patellofemoral pain will be selected and these will be divided into groups A and B respectively. Subsequently, the subjects in group A will be allocated into three groups: Group C (running retraining focused on impact); Group D (Running retraining focusing on cadence); Group C (Control). Pain will be evaluated using the Visual Analogue Pain Scale, function using the Patellofemoral Disorders Scale and kinematic aspects of the trunk and lower limbs using a two-dimensional movement analysis system. Special and functional tests will also be carried out. The retraining will be carried out through 8 sessions divided into 2 weeks lasting from 15 to 30 minutes, 2 in person and 6 unsupervised. Assessments will take place before, immediately and six months after running retraining. Data will be analyzed using SPSS (Statistical Package for Social Sciences) version 22.0, considering a significance level of $p<0.05$. **Expected results:** It is expected that kinematic and functional factors are associated with patellofemoral pain and that the proposed retraining protocols modify the pain, function and kinematic aspects of runners with patellofemoral pain and that such results are maintained for six months.

1. INTRODUCTION

Running is often requested as an exercise for conditioning, leisure and competition and has had a growing number of fans around the world. The incidence of injuries in this population is high and can vary from 19 to 92% according to the definition chosen to conceptualize injuries in these subjects (SARAGIOTTO et al., 2014; YAMATO et al., 2015).

Sports injuries are highly complex and are the result of the multidirectional connection of different factors, through understanding their interaction, a risk profile can be established and thus reduce the chances of an athlete presenting an injury (BITTENCOURT et al ., 2016).

With regard to evaluation, research is currently focused mainly on interactions between kinematic (pelvis drop; thigh adduction; hip internal rotation; knee angle; ankle angle) and kinetic (peak vertical impact; peak tibial acceleration; average vertical impact rate; instantaneous vertical impact rate) since these are related to the main injuries present in runners (CHEUNG; DAVIS, 2011; CLANSEY et al, 2014; CROWELL; DAVIS, 2011; CROWELL; MILNER ; HAMILL, 2010; DIEBAL; GREGORY; ALITZ, 2011; DIEBAL et al, 2012; ERIKSSON; HALVORSEN; GULLSTRAND, 2011; MESSIER; CIRILLO, 1989; NOEHREN; SCHOLZ; DAVIS, 2011; WILLY; SCHOLZ; DAVIS, 2012). Regarding treatment, an intervention that has been used in recent years and that has shown positive results not only in preventing but also in controlling the symptoms generated by such injuries is running retraining (DIEBAL et al., 2012; ERIKSSON; HALVORSEN ; GULLSTRAND, 2011; NOEHREN et al., 2009; TATE; MILNER, 2017; WILLY; SCHOLZ; DAVIS, 2012).

Running retraining is a treatment modality that aims to modify the biomechanical patterns that may be associated with injuries in runners (AGRESTA; BROWN, 2015). Some studies have already been carried out evaluating the effects of running retraining in runners with patellofemoral pain, the most common strategies demand auditory and/or visual feedback (LEIBBRANDT; LOW, 2017; NOEHREN; SCHOLZ; DAVIS, 2011; WILLY; SCHOLZ; DAVIS, 2012), changes in cadence (BONNACI et al, 2017; DOS SANTOS et al., 2019; ESCULIER et al., 2017) and in the initial contact pattern with the ground (DOS SANTOS et al., 2019; ROPER et al. , 2016), to modulate the previously evaluated kinematic and kinetic aspects.

Such studies found that running retraining programs had positive effects on pain (BONNACI et al., 2017; DOS SANTOS et al., 2019; ESCULIER et al., 2017; LEIBBRANDT; LOW, 2017; NOEHREN; SCHOLZ; DAVIS , 2011; ROPER et al., 2016; WILLY; SCHOLZ; DAVIS, 2012), function (DOS SANTOS et al., 2019; ESCULIER et al., 2017; LEIBBRANDT; LOW, 2017; NOEHREN; SCHOLZ; DAVIS, 2011; WILLY; SCHOLZ; DAVIS, 2012) and biomechanics of runners with patellofemoral pain (ESCULIER et al., 2017; LEIBBRANDT; LOW, 2017; NOEHREN; SCHOLZ; DAVIS, 2011; ROPER et al., 2016; WILLY; SCHOLZ; DAVIS, 2012) and that such results were still maintained for 1 (NOEHREN; SCHOLZ; DAVIS, 2011; ROPER et al., 2016; WILLY; SCHOLZ; DAVIS, 2012), 3 (WILLY; SCHOLZ; DAVIS, 2012) and approximately 6 months (DOS SANTOS et al., 2019; ESCULIER et al., 2017).

Of the studies carried out with gait retraining, only one presented a control group in which no intervention was performed (ROPER et al., 2016) and only two followed the results for a period of 6 months (DOS SANTOS et al., 2019; ESCULIER et al. al., 2017). The programs consisted of 8 sessions over 2 weeks (DOS SANTOS et al., 2019; LEIBBRANDT; LOW, 2017; NOEHREN; SCHOLZ; DAVIS, 2011; ROPER et al., 2016; WILLY; SCHOLZ; DAVIS, 2012) or sessions for 6 (BONNACI et al., 2017) and 8 weeks (ESCULIER et al., 2017).

In this sense, there is a need for clinical trials that assess the effectiveness of gait retraining programs, that have a follow-up period and that propose a retraining model that is closer to clinical practice, since such programs are costly and they demand time on the part of the participant and the therapist. Studies with patellofemoral pain are needed since 40% of treated individuals have symptom recurrence within 1 year and 50% do not fully recover within 5 and 8 years (COLLINS et al., 2013; LANKHORST et al., 2016).

The aim of the study is to verify the effects of two partially supervised running retraining programs on pain, function and kinematics of the lower limbs of runners with patellofemoral pain. Secondarily, to investigate the interaction of different kinematic and functional aspects with the presence of patellofemoral pain in runners.

2. OBJECTIVES

2.1. General objective

To verify the effects of two partially supervised running retraining programs on lower limb pain, function and kinematics in runners with patellofemoral pain.

2.2. Specific objectives

Evaluate the knowledge regarding the running retraining of runners in the city of Goiânia.

To investigate whether kinematic and functional aspects are associated with patellofemoral pain during running in runners.

To investigate whether functional aspects are associated with kinematic aspects during running in subjects with patellofemoral pain.

3. MATERIALS AND METHODS

3.1. Study design

Initially, a cross-sectional study will be carried out to verify the knowledge of runners in Goiânia about gait retraining and to evaluate the interaction between kinematic and functional aspects with the presence of patellofemoral pain. This analysis precedes the clinical trial that aims to evaluate the influence of different gait retraining programs on these aspects. After the initial assessment of the runners' knowledge about running retraining, the subjects according to the inclusion and exclusion criteria explained below will be selected and included in groups A and B. Group A will consist of subjects with patellofemoral pain, while group B will be subjects without patellofemoral pain. For the clinical trial, only the subjects of group A (with patellofemoral pain) will be included, these will be divided into three subgroups called group C (Running retraining focused on impact), group D (Running retraining focused on cadence) and control. This second moment of the research will be a Randomized Controlled Clinical Trial (1:1:1), blind, with a follow-up of 6 months, registered in the REBEC (Brazilian Register of Clinical Trials) and carried out in accordance with the recommendations of CONSORT (Consolidated Standards of Reporting Trials) (SCHULZ et al , 2010). The study will be submitted for consideration by the Research Ethics Committee (CEP). The sample size was calculated considering the clinical trial. The sample calculation was performed using the G*Power software, version 3.1, through a paired analysis, considering an alpha of 95%, Power of 95%, effect size of 1.51 and the pain variable (FAUL et al, 2009) . The determined sample was of 7 subjects, considering 10% of sample loss, the final sample will be of 8 subjects in each group (C x D x Control). The sample calculation was performed according to the study by Neal et al., (2018) who evaluated the feasibility of a running retraining program focusing on cadence and found a difference of 2.1 points on the Visual Analog Scale between mean pain before and after program and effect size (Cohen's *d) of 1.7.* In addition to the 24 subjects with patellofemoral pain (8 in each group), another 24 subjects without patellofemoral pain matched according to anthropometric characteristics will be recruited. Study recruitment will occur after approval by the Research Ethics Committee (CEP) and obtaining the clinical trial registration through the Brazilian Registry of Clinical Trials (REBEC).

3.2. Local

This study will be carried out at Instituto Trata located at Rua T-53, 1043 – Setor Bueno, Goiânia, Goiás, CEP: 74215-150

3.3 Inclusion Criteria

For the cross-sectional analysis, participants who have patellofemoral pain and who do not have patellofemoral pain will be included. The inclusion criteria for those with patellofemoral pain will be: subjects of both sexes, rearfoot strike runners with a cadence of less than 170 steps per minute, aged between 18 and 45 years, who present anterior knee pain above 3 points on the Visual Analog Scale (VAS) during running and in at least one activity between crouching, going up and down stairs, kneeling and extending the knee in a resisted way, and that they train/run with an average speed between 10-12 Km/hour. Inclusion criteria for those who do not have patellofemoral pain will be: runners with the same gender and age criteria as the previous group and without any symptoms related to patellofemoral pain.

3.4. Exclusion Criteria

Subjects with patellofemoral pain will be excluded from the clinical trial if they have other diseases in the lower limbs, surgical history in the last year, and if they are not interested in undertaking a 2-week retraining program.

3.5. Randomization

Regarding the clinical trial, participants with patellofemoral pain will be informed that they can be allocated into one of the three study groups and thus "participate in the retraining protocol focused on impact", "participate in the retraining protocol focused on cadence" or "not participate in the running retraining protocol".

A simple randomization sequence will be generated in Microsoft Excel software by one of the study investigators who will not be directly involved in the assessments or treatment. After the initial evaluations, the therapist will open the randomization envelope and the participants will be allocated in one of the three study groups: Group C (Running retraining focused on impact); Group D (Running retraining focusing on cadence); Control.

The allocation will be concealed by means of opaque, sealed and consecutively numbered envelopes. Retraining protocols will be performed immediately after allocation.

3.6. Blinding

Regarding the clinical trial, the outcome evaluator and the investigator performing the statistical analyzes of the data will be blinded to the study participants' allocation group. Due to the nature of the interventions, it will not be possible for the therapist and participants to be blind to treatment conditions.

3.7. Follow-up

Assessments will be made prior to randomization, immediately after running retraining protocols, and six months after completion of study interventions. All assessments will be conducted in person. Between the end of the protocol and the evaluation six months later, monthly messages will be sent asking the participant about the frequency, volume and intensity of training, as well as discomfort during training.

3.8. Instruments

The following instruments will be used for data collection:

- **Consent Form:** contains information in a simple and summarized form, about the title, objectives, methodology, benefits and risks of the study, confidentiality of data and images, the voluntary participation of the subject and the guarantee that he can withdraw from the study at any time.

- **Epidemiological questionnaire:** will be used to characterize the sample and assess knowledge about gait retraining. It contains the following information: name, age (years), body mass (kilograms), height (meters), Body Mass Index (kilograms per square meter), pathological and surgical history, running characteristics (time practicing the sport, speed /pace, volume, frequency, races/year) and questions regarding running retraining.

- **Visual Analog Pain Scale (VAS):** will be used to measure pain intensity. It consists of a numerical scale from 0 to 10 points, where 0 means no pain and 10 means the maximum amount of pain ever experienced by the subject (PIMENTA, 1994).

- **Patellofemoral Disorders Scale:** will be used to assess function. It consists of a questionnaire translated and validated into Portuguese, which contains 13 questions that assess the severity of symptoms and limitation in different activities related to patellofemoral pain. It presents a score between 0 and 100 where the lower the score, the worse the function (AQUINO et al, 2011).

- **Movement Analysis System:** will be used to evaluate the kinematics of the lower limbs. The evaluation will take place through digital videos using two webcams (MyoVideo 139 HD Color Webcam) sampling at 30 frames per second and two LEDs (LED Floodlight). Reflective markers (19 mm) will be placed on the manubrium of the sternum and bilaterally on the anterior superior iliac spine, greater trochanter, lateral epicondyle of the femur, head of the fibula and lateral malleolus (DINGENEN et al., 2018a). All participants will be instructed to run at 10-12 km/hour on a motorized treadmill (Movement XL 1600). A 6-minute acclimatization period on the treadmill will be used before evaluating the running kinematics (LAVCANSKA; TAYLOR; SCHACHE, 2005) . The frontal plane camera will be placed on a portable tripod perpendicular to the frontal plane, at a height of 1.05m and at a distance of 2.0m from the treadmill (DINGENEN et al., 2018a). The sagittal plane camera will be placed on a portable tripod, perpendicular to the sagittal plane, at a height of 0.80 m and at a distance of 1.40 m from the treadmill (DINGENEN et al., 2018a). The videos will be analyzed using the software MyoResearch 3.14 - MyoVideo (Noraxon USA Inc.). In the frontal plane, the angles evaluated will be: contralateral pelvic drop; hip adduction (angles assessed during medium stance) (DINGENEN et al., 2018a; DINGENEN et al., 2018b; MAYKUT et al., 2015). In the sagittal plane, the angles evaluated will be: foot inclination; tibial tilt; ankle dorsiflexion; knee flexion (the first two will be evaluated during initial contact and the last two in medium support) (DINGENEN et al., 2018a; PIPKIN et al., 2016; SOUZA, 2016). In the frontal plane, the deepest landing position will be visually determined by slowly advancing the video frame by frame (DINGENEN et al., 2018a; DINGENEN et al., 2018b; MAYKUT et al., 2015). Midstance in the sagittal plane will be defined visually the same as in the frontal plane and is typically the point where the swing leg crosses the stance leg. Initial contact will be determined visually by slowly advancing the video, frame by frame, and will be defined as the first time the foot touches the ground (PIPKIN et al.,

2016). To analyze the proposed angles, seven steps will be considered (DINGENEN et al., 2018a).

- **PHAST (Physiotherapy Assessment Tool):** will be used to assess the functional aspects (passive stiffness of the hip lateral rotators, isometric strength of the lateral hip rotators, isometric strength of the hip abductors and ankle dorsiflexion range of motion in closed kinetic chain). It is an application that brings together several tests with specific objectives. The aspects evaluated and the form of evaluation are described below:

Stiffness of the hip lateral rotators: The participant is positioned in ventral decubitus with the knee flexed at 90°, the researcher passively moves the lower limb to be tested in internal rotation of the hip. With an inclinometer positioned 5 centimeters below the anterior tuberosity of the tibia, the record in degrees of the amplitude of hip medial rotation will be recorded (CARVALHAIS et al, 2011).

Isometric hip abductor dynamometry: The participant is positioned in lateral decubitus on a stretcher with the upper limbs positioned in front of the body. A stabilizing band is used to fix the trunk and another, positioned five centimeters above the knee joint interline, used to limit the range of motion of hip abduction. The participant will perform a maximum isometric abduction with the tested lower limb for five seconds. This procedure will be performed three times with an interval of fifteen seconds between each isometric contraction. During the test, verbal encouragement will be given to ensure that the participant performs the maximum contraction. The muscle function of the hip abductors in Nm/Kg was recorded using a manual dynamometer (Hand Held –Microfet2 ®) positioned five centimeters above the knee joint interline.

Isometric dynamometry of the hip lateral rotators: The participant is positioned in a seated position, on a stretcher with the upper limbs crossed at chest level. A stabilizing band is used to fix the pelvis in order to minimize trunk movements. The participant will perform maximum isometric hip lateral rotation with the tested lower limb for five seconds. This procedure will be performed three times with an interval of fifteen seconds between each isometric contraction. During the test, verbal encouragement will be given to ensure that the participant performs the maximum contraction. The muscle function of the hip lateral rotators in Nm/Kg is recorded using a manual dynamometer (Hand Held –Microfet2 ®) positioned five centimeters above the medial malleolus of the limb to be evaluated.

Lunge test: The participant is positioned standing, trunk aligned to a wall with the lower limb to be tested in front. Without raising the calcaneus of the posterior lower limb, it must actively flex the knee of the anterior lower limb until it touches the wall. With an inclinometer positioned 15 centimeters below the anterior tuberosity of the tibia, the record in degrees of ankle dorsiflexion amplitude in CCF will be made (BENNELL et al, 1998).

3.9. Protocol

Group C (Running retraining focused on impact): the running retraining protocol will be performed four times a week, with a gradual duration from 15 to 30 minutes, for two weeks, with two sessions supervised and the other six sessions unsupervised, supervised sessions will be the first and fifth sessions. During the supervised sessions, the participants will run in shoes (sneakers they usually wear) at a speed of 10-12 km/hour (training/comfortable speed) and will receive visual feedback (tibial acceleration captured by means of an accelerometer - Tgforce v2.0.0.10) and verbal (commands given by the researchers) throughout the intervention period. The accelerometer will be placed on the anteromedial aspect of the distal region of the tibia (CROWEL; DAVIS, 2011). A television positioned in front of the treadmill will show a graph of tibial acceleration in real time captured by the accelerometer. On the screen, the participant will see a line that represents approximately 50% of the mean peak tibial acceleration obtained during the last minute of running (CROWEL et al., 2010; CROWEL; DAVIS, 2011). The examiner will guide the participant to run smoother, make the steps more silent and keep the tibial acceleration peak below the line shown on the screen (CREABY; SMITH, 2015; CROWEL et al., 2010; CROWEL; DAVIS, 2011) . These instructions will be given during the first minute of retraining and reinforced every 30 seconds according to the need observed by the examiner (CREABY; SMITH, 2015). Participants will be instructed to maintain the new running pattern during the remaining unsupervised sessions held in a location of their choice (CHAN et al., 2018).

Group D (Running retraining focused on cadence): the running retraining protocol will be performed four times a week, with a gradual duration from 15 to 30 minutes, for two weeks, with two sessions supervised and the other six sessions unsupervised, supervised sessions will be the first and fifth sessions. During supervised sessions, participants will run

in shoes (sneakers they usually use) at a speed of 10-12 km/hour (training/comfortable speed), receive guidance regarding their cadence and will run with the help of a metronome with a cadence set between 7.5 to 10%. During the other sessions, the participants will have to perform the running retraining identically to the one performed in the supervised session in a place of their choice.

To ensure that participants in groups C and D carry out the protocols and the way they should be carried out, a supervised session was initially carried out. Other measures adopted by the researchers are: (I) guidance after the supervised session on how unsupervised sessions should take place; (II) delivery of a table showing how unsupervised sessions should take place (see table 1); (III): daily text message on the participants' phone reminding them to carry out the sessions as previously instructed.

Table 1. Protocol that will be performed by the study participants

Week	Day	type of training	feedback time	training time
1	1	Supervised	15 minutes	15 minutes
	2	unsupervised	18 minutes	18 minutes
	3	unsupervised	21 minutes	21 minutes
	4	unsupervised	24 minutes	24 minutes
2	5	Supervised	21 minutes	27 minutes
	6	unsupervised	15 minutes	30 minutes
	7	unsupervised	9 minutes	30 minutes
	8	unsupervised	3 minutes	30 minutes

Source: Authors themselves.

Control Group: will not receive any running retraining strategies or guidance until the end of the study. After this step, the participants will receive guidance relevant to their condition based on the previously performed biomechanical assessments.

3.10. Procedures

Initially, the researchers will publicize the study through pamphlets in Goiânia running advisors, social media and running events held in the city of Goiânia. During the first contact, the objectives and procedures of the study will be clarified and those who agree and

sign the TCLE will answer the epidemiological questionnaire. Those who fit the inclusion and exclusion criteria will be distributed in the cross-sectional analysis groups.

After this stage, the subjects will be invited to the Instituto Trata where pain, function, lower limb kinematics and functional tests will be evaluated. After performing such evaluations, the subjects in group A will be randomized into subgroups C, D and control. Participants in groups C and D will be scheduled for running retraining activities that will take place in the two weeks following the initial assessments. The runners allocated in the control group will continue in the two subsequent weeks performing their training activities without interference from the researchers. After this period, pain, function, lower limb kinematics and functional tests will be evaluated again.

The subjects who performed the evaluations and completed the running retraining protocol will be followed for a period of six months, at the end of which a new reassessment of the aforementioned aspects will be performed in order to observe the short-term effects of the protocol used in the study.

3.11. Statistical analysis

Data analysis will be performed using SPSS (Statistical Package for Social Sciences), version 23.0. Initially, a descriptive analysis will be performed with the calculation of mean, standard deviation and 95% confidence interval for quantitative variables and frequency and percentage for qualitative variables.

To assess whether the kinematic and functional aspects are associated with patellofemoral pain, a linear regression will be performed considering the presence of patellofemoral pain as a dependent variable and the kinematic and functional aspects as independent variables. A linear regression will also be carried out considering the kinematic aspects as dependent variables and the functional aspects as independent variables, so that all the specific objectives of the project are answered.

To verify the normality of the data, the Shapiro-Wilk test will be used. To compare the variables before, immediately and six months after the running retraining program in the study groups, the repeated measures ANOVA (parametric data) or the Friedman test (non-parametric data) will be used. To compare the variables between the different study groups, ANOVA (parametric data) or Kruskall-Wallis (non-parametric data) will be used. To make

comparisons between pairs, Tukey's post-hoc test (parametric data) or the Wilcoxon/Mann Whitney U test with Bonferroni correction will be used. A significance level of $p<0.05$ will be used. An intention-to-treat analysis will be performed where subjects will be analyzed regardless of protocol completion. If there is any participant who does not complete the same, the reasons will be described in the manuscript results section.

BIBLIOGRAPHIC REFERENCES

AGRESTA, C.; BROWN, A. Gait retraining for injured and healthy runners using augmented feedback: a systematic literature review. **Journal of Orthopedic & Sports Physical Therapy** , v. 45, no. 8, p. 576-584, 2015.

AQUINO, V. et al. Translation and cultural adaptation of the Scoring of Patellofemoral Disorders questionnaire into Portuguese : a preliminary study. **Acta Ortopédica Brasileira** , v. 19, no. 5, 2011.

BENNELL, K et al. Intra-rater and inter-rater reliability of a weight-bearing lunge measure of ankle dorsiflexion. **Australian Journal of Physiotherapy,** v. 44, no. 3, p. 175-180, 1998.

BITTENCOURT, NFN et al. Complex systems approach for sports injuries: moving from risk factor identification to injury pattern recognition—narrative review and new concept. **British Journal of Sports Medicine** , v. 50, no. 21, p. 1309-1314, 2016.

BONACCI, J. et al. Gait retraining versus foot orthoses for patellofemoral pain: a pilot randomized clinical trial. **Journal of science and medicine in sport** , v. 21, no. 5, p. 457-461, 2018.

CARVALHAIS, VO; DE ARAUJO, VL; SOUZA, T. R et al. Validity and reliability of clinical tests for assessing hip passive stiffness. **Manual Therapy,** v. 16, no. 3, p.240-245, 2011.

CHAN, ZY S et al. Gait retraining for the reduction of injury occurrence in novice distance runners: 1-year follow-up of a randomized controlled trial. **The American Journal of Sports Medicine** , v. 46, no. 2, p. 388-395, 2018.

CHEUNG, RT; DAVIS, IS Landing pattern modification to improve patellofemoral pain in runners: a case series. **Journal Orthopedic and Sports Physical Therapy** , v. 41, p. 914-919, 2011.

CLANSEY, AC; HANLON, M.; WALLACE, E.S et al . Influence of tibial shock feedback training on impact loading and running economy. **Medicine Science and Sports Exercise** , v. 46, p. 973-981, 2014.

COLLINS, NJ et al. Prognostic factors for patellofemoral pain: a multicentre observational analysis. **British Journal of Sports Medicine,** v. 47, no. 4, p. 227-233, 2013.

CREABY, MW; SMITH, MM Franettovich. Retraining running gait to reduce tibial loads with clinician or accelerometry guided feedback **. Journal of science and medicine in sport** , v. 19, no. 4, p. 288-292, 2016.

CROWELL, HP; DAVIS, IS Gait retraining to reduce lower extremity loading in runners. **Clinical Biomechanics (Bristol, Avon),** v. 26, p. 78-83, 2011

CROWELL, HP; MILNER, CE; HAMILL, J et al. Reducing impact loading during running with the use of real-time visual feedback. **Journal of Orthopedic Sports Physical Therapy,** v. 40, p. 206-213, 2010.

DIEBAL, AR; GREGORY, R.; ALITZ, C. Effects of forefoot running on chronic exertional compartment syndrome: a case series. **International Journal of Sports Physical Therapy** , v. 6, p. 312-321, 2011.

DIEBAL, AR; GREGORY, R.; ALITZ, C et al. Forefoot running improves pain and disability associated with chronic exertional compartment syndrome. **American Journal of Sports Medicine,** v. 40, p. 1060-1067, 2012.

DINGENEN, B. et al. Test-retest reliability of two-dimensional video analysis during running. **Physical Therapy in Sport** , v. 33, p. 40-47, 2018a.

DINGENEN, B. et al. Are two-dimensional measured frontal plane angles related to three-dimensional measured kinematic profiles during running? **Physical Therapy in Sport** , v. 29, p. 84-92, 2018b.

DOS SANTOS, AF et al. Effects of three gait retraining techniques in runners with patellofemoral pain. **Physical Therapy in Sport** , v. 36, p. 92-100, 2019.

ERIKSSON , M.; HALVORSEN, KA; GULLSTRAND, L. Immediate effect of visual and auditory feedback to control the running mechanics of well-trained athletes. **Journal of Sports Science** , vol. 29, p. 253-262, 2011.

ESCULIER, Jean-Francois. et al. Is combining gait retraining or an exercise program with education better than education alone in treating runners with patellofemoral pain? A randomized clinical trial. **British Journal of Sports Medicine** , v. 52, no. 10, p. 659-666, 2018.

HEIDERSCHEIT, B. Always on the run. **Journal of Orthopedic and Sports Physical Therapy** , v. 44, no. 10, 2014.

HESPANHOL JUNIOR, L. C et al. Health and economic burden of running-related injuries in runners training for an event: A prospective cohort study. **Scandinavian journal of medicine & science in sports,** v. 26, no. 9, p. 1091-1099, 2016.

FAUL, F.; ERDFELDER, E.; BUCHNER, A et al. Statistical power analyzes using G*Power 3.1: Tests for correlation and regression analyses. **Behavior Research Methods** , v. 41, no. 4, p. 1149-1160, 2009.

LANKHORST, NE et al. Factors that predict a poor outcome 5–8 years after the diagnosis of patellofemoral pain: a multicentre observational analysis. **British Journal of Sports Medicine** , v. 50, no. 14, p. 881-886, 2016.

LAVCANSKA, V.; TAYLOR, NF; SCHACHE, AG Familiarization to treadmill running in young unimpaired adults. **Human movement science** , v. 24, no. 4, p. 544-557, 2005.

LEIBBRANDT, DC; LOUW, QA Targeted Functional movement retraining to improve pain, function, and biomechanics in subjects with anterior knee pain: A case series. **Journal of sport rehabilitation,** vol. 27, no. 3, p. 218-223, 2018.

MAYKUT, JN et al. Concurrent validity and reliability of 2d kinematic analysis of frontal plane motion during running. **International journal of sports physical therapy** , v. 10, no. 2, p. 136, 2015.

MESSIER, SP; CIRILLO, KJ Effects of a verbal and visual feedback system on running technique, perceived exertion and running economy in female novice runners. **Journal of Sports Science, v** . 7, p. 113-126, 1989.

NEAL, BS et al. The effects & mechanisms of increasing running step rate: A feasibility study in a mixed-sex group of runners with patellofemoral pain. **Physical Therapy in Sport** , v. 32, p. 244-251, 2018.

NOEHREN, BW; DAVIS, I. Can Gait Retraining Change Hip Kinematics And Reduce Symptoms In Runners With Patellofemoral Pain Syndrome? 604May 27 2:15 PM-2:30 PM. **Medicine & Science in Sports & Exercise** , v. 41, no. 5, p. 26, 2009.

NOEHREN, B.; SCHOLZ, J.; DAVIS, I. The effect of realtime gait retraining on hip kinematics, pain and function in subjects with patellofemoral pain syndrome. **British Journal of Sports Medicine** , v. 45, p. 691-696, 2011.

PIMENTA, CAM **Pain assessment scales** . In: TEIXEIRA, M. D (ed.) Pain general concepts. São Paulo: Limay, 1994.

PIPKIN, A. et al. Reliability of a qualitative video analysis for running. **Journal of orthopedic & sports physical therapy** , v. 46, no. 7, p. 556-561, 2016.

ROPER, JL et al. The effects of gait retraining in runners with patellofemoral pain: A randomized trial. **Clinical biomechanics,** v. 35, p. 14-22, 2016.

SARAGIOTTO, B. I et al. What are the main risk factors for running-related injuries? **Sports medicine** , v. 44, no. 8, p. 1153-1163, 2014.

SCHULZ, KF; ALTMAN, DG; MOHER, D.; FOR THE CONSORT GROUP. CONSORT 2010 Statement: updated guidelines for reporting parallel group randomized trials. **Annals of Internal Medicine,** 2010;152.

SOUZA, RB An evidence-based videotaped running biomechanics analysis. **Physical medicine and rehabilitation clinics** , v. 27, no. 1, p. 217-236, 2016.

TATE, J. J.; MILNER, CE Sound-intensity feedback during running reduces loading rates and peak impact. **Journal of Orthopedic & Sports Physical Therapy** , v. 47, no. 8, p. 565-569, 2017.

WILLY, RW; SCHOLZ, JP; DAVIS, IS Mirror gait retraining for the treatment of patellofemoral pain in female runners. **Clinical Biomechanics (Bristol, Avon)** , v. 27, p. 1045-1051, 2012.

YAMATO, TP; SARAGIOTTO, BT; LOPES, AD A consensus definition of running-related injury in recreational runners: a modified Delphi approach. **Journal of Orthopedic & Sports Physical Therapy** , v. 45, no. 5, p. 375-380, 2015.
